# Enhancing Gasdermin-induced tumor pyroptosis through preventing ESCRT-dependent cell membrane repair augments antitumor immune response

**DOI:** 10.1038/s41467-022-34036-8

**Published:** 2022-10-24

**Authors:** Zhaoting Li, Fanyi Mo, Yixin Wang, Wen Li, Yu Chen, Jun Liu, Ting-Jing Chen-Mayfield, Quanyin Hu

**Affiliations:** 1 Pharmaceutical Sciences Division, School of Pharmacy, University of Wisconsin-Madison, Madison, WI 53705 USA; 2 Carbone Cancer Center, School of Medicine and Public Health, University of Wisconsin-Madison, Madison, WI 53705 USA; 3 Wisconsin Center for NanoBioSystems, School of Pharmacy, University of Wisconsin-Madison, Madison, WI 53705 USA

**Keywords:** Tumour immunology, Immunotherapy, Tumour immunology

## Abstract

Pore-forming Gasdermin protein-induced pyroptosis in tumor cells promotes anti-tumor immune response through the release of pro-inflammatory cytokines and immunogenic substances after cell rupture. However, endosomal sorting complexes required for transport (ESCRT) III-mediated cell membrane repair significantly diminishes the tumor cell pyroptosis by repairing and subsequently removing gasdermin pores. Here, we show that blocking calcium influx-triggered ESCRT III-dependent membrane repair through a biodegradable nanoparticle-mediated sustained release of calcium chelator (EI-NP) strongly enhances the intracellularly delivered GSDMD-induced tumor pyroptosis via a bacteria-based delivery system (VNP-GD). An injectable hydrogel and a lyophilized hydrogel-based cell patch are developed for peritumoral administration for treating primary and metastatic tumors, and implantation for treating inoperable tumors respectively. The hydrogels, functioning as the local therapeutic reservoirs, can sustainedly release VNP-GD to effectively trigger tumor pyroptosis and EI-NP to prevent the ESCRT III-induced plasma membrane repair to boost the pyroptosis effects, working synergistically to augment the anti-tumor immune response.

## Abstract (web summary)

Activation of ESCRT-mediated cell membrane repair can reduce the extent of tumor cell pyroptosis. Here the authors develop two formulations (an injectable hydrogel and a cell patch) for the sustained release of a Gasdermin-D bacteria-based delivery system and of biodegradable nanoparticles loaded with an ESCRT inhibitor, triggering pyroptosis and antitumor immune responses in preclinical cancer models.

## Introduction

Pyroptosis, a type of programmed cell death mediated by Gasdermin proteins, is characterized by the continuous expansion of cells forming large ballooning bubbles until the cell membrane ruptures, resulting in the release of cellular contents and subsequent activation of a strong inflammatory response^1,2^. As an important innate immune response in the body, pyroptosis plays a crucial role in antagonizing infection and endogenous danger signals^3,4^. Moreover, the latest research reveals that cytotoxic lymphocytes rely on Gasdermin-mediated pyroptosis to kill tumor cells, suggesting that pyroptosis is also closely involved in anticancer immune response and rising as a very promising method for cancer treatment. Specifically, Granzyme A released from cytotoxic lymphocytes cleaves Gasdermin B (GSDMB) to trigger pyroptosis in target tumor cells, while cytotoxic lymphocytes secreted Granzyme B or chemotherapy-induced activated caspase 3 cleaves Gasdermin E (GSDME) to trigger tumor pyroptosis^5–7^. In addition, activated caspase 8 could cleave Gasdermin C (GSDMC) to trigger tumor pyroptosis. Moreover, the working mechanism of Gasdermin A (GSDMA) in tumor pyroptosis is unclear, but it was found that GSDMA could also trigger tumor pyroptosis and exhibit antitumor efficacy^8^. Among all Gasdermin family proteins, membrane perforin Gasdermin D (GSDMD) acts as an effective executor of pyroptosis mainly in immune cells, while its function and working mechanism in tumor cells remain elusive^9,10^. Notably, there is increasing evidence showing that pyroptosis can inhibit cancer cell proliferation by inducing inflammatory cell death^9^. In-depth study of the mechanism of tumor pyroptosis will provide new directions and molecular targets for cancer immunotherapy. Nowadays, the response rate of clinical cancer immunotherapy, including the well-known immune checkpoint blockade therapy remains very low, especially for nonimmunogenic tumors^11–14^. Therefore, leveraging pyroptosis-mediated antitumor immune response to overcome immunosuppression would be a promising strategy for future cancer immunotherapy.

Despite the encouraging progress of pyroptosis-mediated cancer immunotherapy, there are still emerging limitations restricting its wider application. First, the expression of Gasdermins in cancer tissue is suppressed by DNA methylation. For example, methylation of DFNA5 (deafness autosomal dominant 5) gene reduces the GSDME expression in most tumor tissues, thus restricting T cell- or NK cell-mediated cytotoxicity to tumor cells^15^. Furthermore, even if there is the expression of full-length Gasdermins in tumor cells, pyroptosis cannot occur when the associated caspase signaling pathway is not activated in the tumor cells^16^. More importantly, calcium influx-triggered assembly of the endosomal sorting complexes required for transport (ESCRT) III system could prevent the cell from programmed cell death and further cell lysis by facilitating damaged cell membrane repair, which significantly dampens the tumor pyroptosis. Therefore, guaranteeing sufficient Gasdermin intracellular delivery, activating the related caspase signaling pathway, and preventing ESCRT-mediated cell membrane repair are progressive prerequisites for the realization and enhancement of pyroptosis-mediated cancer immunotherapy.

**Fig. 1:**
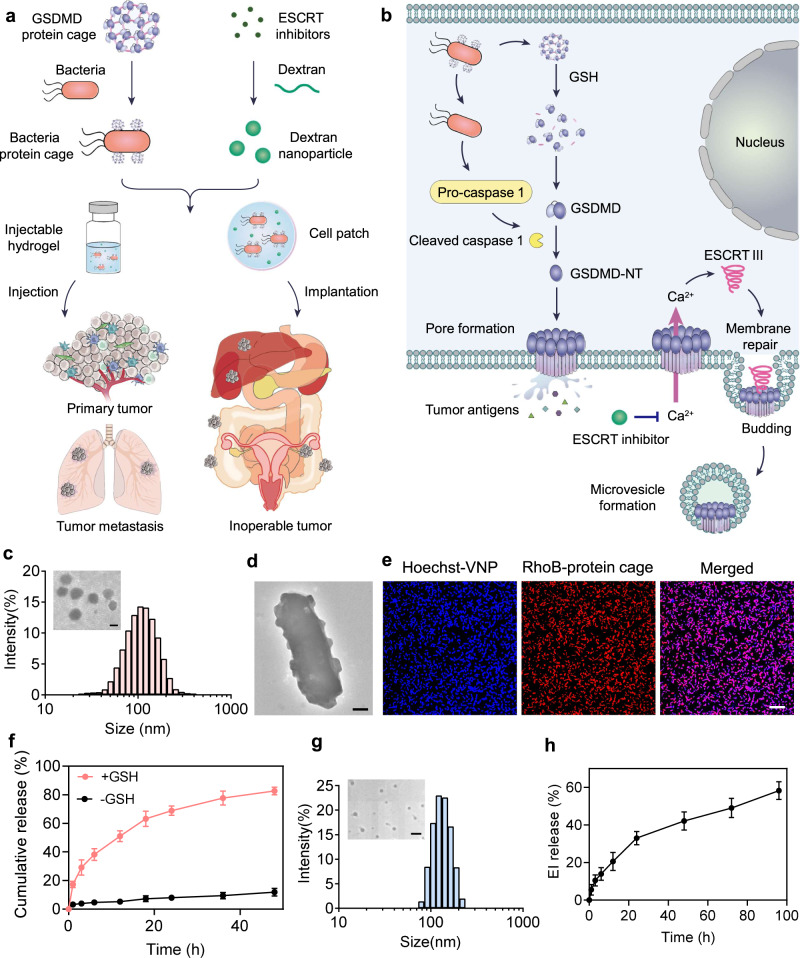
Working mechanism and preparation and characterization of the hydrogel-based bacteria protein cage delivery system. **a** The GSDMD proteins were crosslinked into protein cages and then conjugated on the surface of attenuated *Salmonella typhimurium* (designated VNP-GD). The ESCRT inhibitor was loaded in the dextran nanoparticles (designated EI-NP). Two formulations, an injectable hydrogel and a cell patch, were developed to co-load VNP-GD and EI-NP to treat primary tumors through local administration and inoperable cancer through implantation. **b** The underlying mechanism of tumor pyroptosis triggered by VNP-GD and further enhanced by EI-NP. Firstly, after the invasion of the VNP-GD into the tumor, the GSDMD protein would be released upon GSH stimulation, and the abundant flagella on the surface of bacteria could activate the caspase 1 into cleaved caspase 1, which will further cleave the GSDMD protein to the N-terminal GSDMD that will multimerize and perforate the cell membrane, initiating cell pyroptosis. Secondly, the released ESCRT inhibitor from EI-NP could effectively block the calcium influx to inhibit the ESCRT III-mediated membrane repair to enhance the tumor pyroptosis. **c** Particle size and transmission electronic microscope (TEM) image (inserted) of the protein cages (scale bar = 100 nm). **d** Representative TEM image of the protein cage-conjugated VNP bacteria (VNP-GD, scale bar = 200 nm). The experiments were repeated three times independently. **e** Confocal images of the conjugation of the protein cage (labeled with Rhodamine B) on the surface of the VNP bacteria (labeled with Hoechst), scale bar = 10 μm. The experiments were repeated three times independently. **f** Cumulative release of the protein from the bacteria protein cage with or without the trigger of GSH (10 mM). Data are presented as mean ± s.d. (*n* = 3 biologically independent samples). **g** Particle size and transmission electronic microscope (TEM) image (inserted) of the EI-NP (scale bar = 500 nm). **h** ESCRT inhibitor release profile from the dextran nanoparticle (EI-NP) at predetermined time points. Data are presented as mean ± s.d. (*n* = 3 biologically independent samples). Source data are provided as a Source Data file.

In this work, to leverage Gasdermin-triggered pyroptosis for antitumor immunotherapy, we develop an intracellular bacterium—attenuated *Salmonella typhimurium* (VNP) delivery system to shuttle Gasdermin D protein to initiate the tumor cell pyroptosis. To facilitate the VNP modification and further intracellular release of GSDMD, GSDMD proteins are assembled into protein nanocages by crosslinking proteins through bifunctional glutathione (GSH)-responsive linkers. GSDMD protein cage-conjugated VNP bacteria (designated VNP-GD) could effectively shuttle GSDMD to the cytoplasm of the tumor cells, which will subsequently release GSDMD intracellularly upon the activation of elevated GSH concentration to trigger tumor cell pyroptosis (Fig. 1a). To overcome the plasma membrane repair mediated by ESCRT III machinery, we further encapsulate an ESCRT inhibitor (a Ca^2+^ chelator BAPTA-AM) into a biodegradable dextran nanoparticle (designated EI-NP) for sustained release of BAPTA-AM to prevent Ca^2+^ influx-mediated recruitment of ESCRT machinery to the damaged cell membrane (Fig. 1b). Finally, to enable the in vivo treatment, we develop two formulations (an injectable hydrogel and a cell patch) to co-load VNP-GD and EI-NP for primary and metastatic tumors and unresectable tumor treatments (Fig. 1a). We find that in the metastatic breast cancer model, melanoma model and inoperable ovarian cancer model, the tumor cell pyroptosis is triggered by VNP-GD delivery systems, leading to substantial programmed necrotic tumor cells and strong antitumor immune response, which are further enhanced by preventing ESCRT-mediated cell membrane repair. Furthermore, this treatment strategy can work synergistically with immune checkpoint blockade therapy to improve the immunotherapy efficacy against multiple immunogenic and non-immunogenic tumor models, paving a promising way to potentially increase the objective response rate of immune checkpoint inhibition in the clinic.

## Results

### Preparation and characterization of the hydrogel-based bacteria protein cage delivery system

Cells tend to maintain redox homeostasis. The oxidative stress in tumor tissue usually causes higher GSH expression in tumor cells than that in normal cells^17^. Therefore, to achieve selective protein release inside tumor cells, we first synthesized a GSH responsive linker (Supplementary Fig. 1) to crosslink GSDMD proteins to form protein cages (designated GD) that can be easily modified on the surface of VNP. Furthermore, as shown in Fig. 1c and Supplementary Fig. 2, GSDMD proteins were assembled into protein cages with the particle size of about 130 nm and the zeta potential of about −7.7 mV, through crosslinking with GSH responsive linkers. Typically, negatively charged free GSDMD proteins or protein cages are hard to transport into tumor cells to initiate pyroptosis. In this regard, VNP20009 (VNP) was selected as a carrier to shuttle GSDMD to the cytoplasm of tumor cells. VNP is a genetically modified strain of attenuated *Salmonella typhimurium* with a good biosafety profile^18,19^. As an intracellular bacterium with flagellum to enable strong mobility and natural tropism toward tumor hypoxic areas, VNP is an excellent carrier for tumor-selective and intracellular delivery of protein cargoes^20^. Moreover, the abundant flagella of VNP could activate the caspase 1 to transform the delivered GSDMD proteins into the active form for triggering tumor cell pyroptosis. After removing extra linkers by ultrafiltration, the GSDMD protein cages were conjugated on the surface of VNP by a facile amide reaction^21^. The obvious particle protrusions were visualized on the surface of the modified bacteria under transmission electron microscope (TEM) imaging (Fig. 1d, Supplementary Fig. 3) compared to the unmodified bacteria (Supplementary Fig. 4). Furthermore, the successful conjugation was verified by the colocalization of the Hoechst-labeled bacteria and Rhodamine B-labeled protein cages (Fig. 1e). According to the flow cytometry assay, over 97% of the bacteria were conjugated with protein cages (Supplementary Fig. 5). Moreover, the successful GSDMD protein cage conjugation was also verified by the western blot assay (Supplementary Fig. 6). In addition, it was demonstrated that protein cages conjugation did not affect the viability of VNP (Supplementary Fig. 7). Subsequently, we evaluated the protein release behavior of the protein cage conjugated bacteria delivery system. The presence of 10 mM GSH (typically representing the intracellular GSH level in tumor cells^22^) significantly increased the protein release rate, with over 60% of protein released within 20 h compared to that without GSH (Fig. 1f). The released protein was substantiated to be GSDMD by western blot assay (Supplementary Fig. 8).

Under normal circumstances, the Ca^2+^ concentration outside the cell is much higher than the intracellular Ca^2+^ concentration, and the maintenance of this concentration gradient is mainly governed by the calcium pump on the cell surface^23^. However, after pyroptosis-induced pore formation and cell membrane damage, the calcium influx would trigger the ESCRT-mediated cell membrane repair^24,25^. To inhibit the calcium influx triggered ESCRT cell membrane repair for improving VNP-GD-induced tumor cell pyroptosis, a biocompatible dextran nanoparticle (EI-NP) loaded with the ESCRT inhibitor (a potent calcium ion antagonist, BAPTA-AM) was formulated, showing the monodispersed spherical structure under TEM observation with a particle size of ~150 nm and the zeta potential of −1.8 mV (Fig. 1g, Supplementary Fig. 2). Moreover, over 58% of the ESCRT inhibitor was released from the dextran nanoparticle within 96 h under PH 6.5 condition that mimics the pH level in the tumor microenvironment^26,27^ (Fig. 1h).

### Characterization of VNP-GD-induced tumor cell pyroptosis

**Fig. 2:**
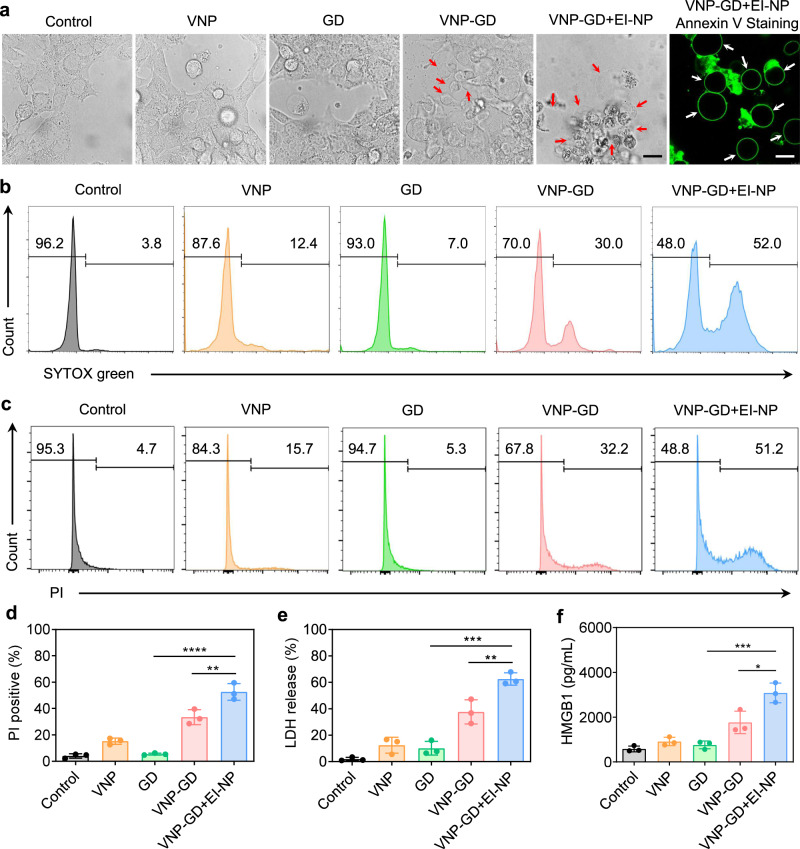
Characterization and verification of VNP-GD-induced tumor cell pyroptosis. **a** Direct observation of the pyroptosis of 4T1 cells under confocal microscope after different treatments with PBS, VNP, GD (GSDMD protein cage), VNP-GD (GSDMD protein cage-conjugated VNP), and VNP-GD+EI-NP (GSDMD protein cage-conjugated VNP + EI-NP) for 24 h. 4T1 cells were further stained with Annexin V for imaging. Scale bar: 15 µm. The experiments were repeated three times independently. Flow cytometry analysis of cell uptake of SYTOX green (**b**) and PI (**c**) in 4T1 tumor cells after incubation with PBS, VNP, GD (GSDMD protein cage), VNP-GD (GSDMD protein cage-conjugated VNP), and VNP-GD+EI-NP (GSDMD protein cage-conjugated VNP + EI-NP) for 24 h. **d** Data analysis of the PI-positive cells after different treatments. Data are presented as mean ± s.d. (*n* = 3 biologically independent samples) and analyzed with one-way ANOVA followed by Dunnett’s multiple comparisons test. VNP-GD + EI-NP vs. VNP-GD: **P = 0.0055; VNP-GD + EI-NP vs. GD: ****P < 0.0001. LDH (**e**) and HMGB1 (**f**) release after the treatments of PBS, VNP, GD (GSDMD protein cage), VNP-GD (GSDMD protein cage-conjugated VNP), and VNP-GD+EI-NP (GSDMD protein cage-conjugated VNP + EI-NP) for 24 h. Data are presented as mean ± s.d. (*n* = 3 biologically independent samples) and analyzed with one-way ANOVA followed by Dunnett’s multiple comparisons test. (**e** VNP-GD+EI-NP vs. VNP-GD: **P = 0.0068; VNP-GD+EI-NP vs. GD: ***P = 0.0001; **f** VNP-GD+EI-NP vs. VNP-GD: *P = 0.0119; VNP-GD + EI-NP vs. GD: ***P = 0.0007). Source data are provided as a Source Data file.

The good biosafety and tumor-targeting tendency of VNP make it an excellent vector to deliver therapeutic proteins directly to tumors^19,28^. As an intracellular bacteria strain, VNP efficiently delivered GD protein cages into 4T1 cells (Supplementary Fig. 9). In addition, as a genetically stable Salmonella typhimurium strain, the bacteria-based delivery system is able to invade the host cell via both a Trigger and a Zipper mechanism. Specifically, the Trigger-dependent mechanism requires a type III protein secretion system (T3SS) based invasion while the Zipper mechanism relied on an Rck protein-based invasion^29^. Moreover, because of its hypoxia tropism and abundant flagella-enabled strong motility^19^, VNP-GD showed excellent tumor penetration ability in the 4T1 3D tumor cell sphere (Supplementary Fig. 10). After invasion into the cells, the higher GSH level in the tumor cytoplasm will break the GSH responsive linkers in the protein cages, triggering the release of GSDMD proteins for subsequent tumor cell pyroptosis. After the verification of the successful intracellular GSDMD protein delivery, next the efficacy of VNP-GD in triggering tumor cell pyroptosis was evaluated in both 4T1 and B16F10 tumor cells. Pyroptosis is typically characterized by the expansion of cells forming large ballooning bubbles before the cell membrane ruptures^30,31^. Thus, the cellular morphology after different treatments was first observed under a confocal microscope, and the transparent large ballooning bubbles forming in 4T1 cells and B16F10 cells were found after the treatments of VNP-GD and VNP-GD+EI-NP (Fig. 2a, Supplementary Fig. 11 and Supplementary Fig. 12). Moreover, blocking ESCRT III-mediated membrane repair by EI-NP substantially increased the number of pyroptotic tumor cells generated by VNP-GD. However, negligible tumor pyroptosis was observed in either the VNP group or GD group, suggesting that the initiation of tumor cell pyroptosis requires the presence of both VNP and GD.

SYTOX green is a nucleic acid dye that can be applied to characterize the cell membrane integrity. It can only pass through damaged cytoplasmic membranes to bind the nucleic acid in which its fluorescence intensity will be significantly enhanced after binding^6^. Therefore, the cell uptake of SYTOX green could serve as an indication of tumor cell pyroptosis after different treatments. It was found that the VNP-GD increased the cell uptake of SYTOX green from 3.8% (control group) to 30%. Notably, VNP-GD+EI-NP treatment significantly enhanced the cell uptake of SYTOX green to 52%, suggesting the strongest pyroptosis-induced cell membrane rupture and pore formation among all treatment groups (Fig. 2b, Supplementary Fig. 13). Moreover, the propidium iodide (PI) staining was performed to further verify the programmed cell death and cell membrane damage. As shown in Fig. 2c, d, the percentage of PI-positive cells in VNP-GD+EI-NP group was 51.2% which was significantly higher than that in the VNP-GD (32.2%), VNP (15.7%), and GD (5.33%) groups. During cell pyroptosis, the activated N-terminal GSDMD proteins would insert into the cell membrane to form the pore, and eventually the cells rupture to release the inside contents, which could result in elevated lactate dehydrogenase (LDH) released from tumor cells^32,33^. Therefore, LDH release was detected to evaluate the cytotoxicity of the VNP-GD+EI-NP against tumor cells. It was found that after blocking the ESCRT-mediated cell membrane repair, the LDH release in VNP-GD+EI-NP group was 1.60-fold higher than that in VNP-GD group (Fig. 2e). Furthermore, as a typical intracellular tumor antigen, the high mobility group box 1 protein (HMGB1) release was also detected by the enzyme-linked immunosorbent assay (ELISA) kit, and the VNP-GD+EI-NP treatment increased HMGB1 expression from 583 pg/ml (control group) to 3083 pg/ml, which was 1.75-fold higher than that of VNP-GD (Fig. 2f), suggesting the enhanced tumor antigen release after the VNP-GD+EI-NP treatment that was attributed to the induced tumor cell pyroptosis.

### Mechanistic study of tumor pyroptosis and ESCRT-dependent cell membrane repair

**Fig. 3:**
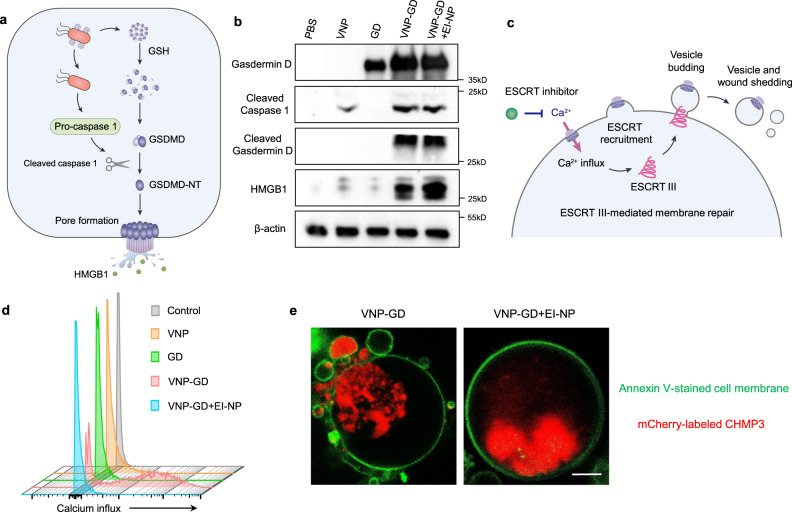
Working mechanism of GSDMD-induced pyroptosis and ESCRT III-mediated cell membrane repair. **a** Schematic illustration of the pyroptosis-related signaling pathway. **b** Western blot assay of the pyroptosis signaling pathway in 4T1 cells after treatments of PBS, VNP, GD (GSDMD protein cage), VNP-GD (GSDMD protein cage-conjugated VNP), and VNP-GD+EI-NP (GSDMD protein cage-conjugated VNP + EI-NP). The experiments were repeated three times independently. **c** Schematic illustration of the calcium influx induced ESCRT III-mediated membrane repair during cancer cell pyroptosis. **d** Flow cytometry assay of the Ca^2+^ influx detection with Fluo-8 AM in 4T1 cells after treatment with PBS, VNP, GD, VNP-GD, and VNP-GD+EI-NP for 24 h (*n* = 3 biologically independent samples). **e** Direct observation of the ESCRT III-mediated 4T1 cancer cell membrane repair during pyroptosis after treatment with VNP-GD and VNP-GD+EI-NP for 24 h (*n* = 4 biologically independent samples). The cell membrane was stained with Annexin V. The 4T1 cells were genetically engineered to express mCherry-labeled charged multivesicular body protein 3 (CHMP 3) protein, which is the main component of ESCRT III machinery. Scale bar: 5 µm. The experiments were repeated three times independently. Source data are provided as a Source Data file.

GSDMD protein-induced pyroptosis signaling pathway is mainly illuminated in immune cells^9^ and remains elusive in tumor cells. To reveal the underlying mechanism of VNP-GD-triggered tumor cell pyroptosis, the western blot assay was first performed to investigate the key protein expressions in the activated tumor cell pyroptosis signaling pathway. As shown in Fig. 3a, b, 4T1 tumor cells without any treatment showed negligible GSDMD protein expression, while VNP-GD could efficiently deliver GSDMD to the cytoplasm of 4T1 cells. Furthermore, it was found that VNP could activate the caspase 1 to cleaved caspase 1, which is probably attributed to the flagella on the surface of the bacteria^34^. However, without the VNP treatment, there is negligible cleaved caspase 1 expression in PBS and GD groups, demonstrating the crucial role of VNP in activating the caspase 1 signaling pathway. Moreover, the immune fluorescence assay demonstrated that VNP also successfully activated cleaved caspase 1 in 4T1 tumors in vivo (Supplementary Fig. 14). Next, the cleaved GSDMD proteins (N-terminal GSDMD) were observed in both VNP-GD and VNP-GD+EI-NP groups, and there was no significant difference between these two groups, indicating that the enhanced tumor cell pyroptosis by EI-NP treatment was not through increasing the cleavage of GSDMD. Moreover, there was GSDMD expression detected in the GD group, but no cleaved GSDMD was detected, which revealed that GSDMD could not be activated to induce further pyroptosis without VNP-mediated caspase 1 cleavage. In addition, selective caspase-1 and pan-caspase inhibitors have been utilized to further demonstrate that caspase-1 is the major driver of GSDMD activation (Supplementary Fig. 15). Subsequently, after the GSDMD cleavage, the N-terminal GSDMD will bind to phospholipid proteins on the cell membrane to form holes to induce pyroptosis, accompanied with the release of intracellular inflammatory antigens. Notably, compared with the VNP-GD group, a substantially increased HMGB1 expression was observed in the VNP-GD+EI-NP group, suggesting stronger cell membrane rupture generated by VNP-GD+EI-NP treatment. Collectively, it was demonstrated here that VNP-GD could efficiently trigger tumor cell pyroptosis by the combination of enhancing intracellular delivery of GSDMD via VNP and further activation of N-terminal GSDMD mediated by VNP-induced caspase 1 cleavage. Moreover, the addition of EI-NP enhanced the pyroptosis as evidenced by the increased generation of tumor antigen HMGB1 but did not alter the expression of cleaved caspase 1 or cleaved GSDMD.

To investigate the EI-NP-mediated pyroptosis enhancement, the ESCRT III machinery-mediated membrane repair was investigated after VNP-GD+EI-NP treatment. The calcium influx, the initiator of ESCRT III-induced membrane repair, was first evaluated to verify if pyroptosis could trigger calcium influx and if EI-NP could inhibit the calcium influx in the 4T1 tumor cells. As shown in Fig. 3c, d and Supplementary Fig. 16, VNP-GD could trigger the calcium influx as indicated by an obvious increase of the fluorescence intensity of the green fluorescent calcium-binding dye (Fluo-8 AM), which was probably attributed to the pyroptosis-induced pore formation or cell membrane damage. However, after treatment with the VNP-GD+EI-NP group, there was no significant increase in the fluorescence intensity compared to the control, VNP and GD groups, suggesting the blocked calcium influx by the EI-NP. Moreover, the role of ESCRT III machinery in tumor cell membrane repair during pyroptosis was investigated with the hypothesis of blocking calcium influx to prevent the recruitment of ESCRT III machinery. Charged multivesicular body protein 3 (CHMP3) is a main functional part of ESCRT III machinery during cell membrane repair^35^, therefore, a mouse full-length-CHMP3-mCherry plasmid was constructed and transfected into 4T1 cells to obtain the CHMP3-mCherry-expressing 4T1 cells. As shown in Fig. 3e and Supplementary Fig. 17, the mCherry-labeled CHMP3 was located on the cell membrane and formed microvesicles to fix the pores and help the cell membrane repair during VNP-GD-triggered tumor pyroptosis. Notably, after the treatment of VNP-GD+EI-NP, the mCherry-labeled CHMP3 proteins were mainly distributed in the cytoplasm and not involved in the cell membrane repair with negligible microvesicles formed during pyroptosis, which was attributed to the prevention of the recruitment of ESCRT III machinery by blocking calcium influx. It was demonstrated that ESCRT III-mediated membrane repair could increase the resistance of 4T1 tumor cells to pyroptosis through sewing the pore, budding and forming microvesicles, thus blocking ESCRT III-mediated membrane repair via preventing calcium influx could enhance tumor pyroptosis.

### Antitumor efficacy in 4T1 metastatic triple-negative breast cancer and B16F10 melanoma

**Fig. 4:**
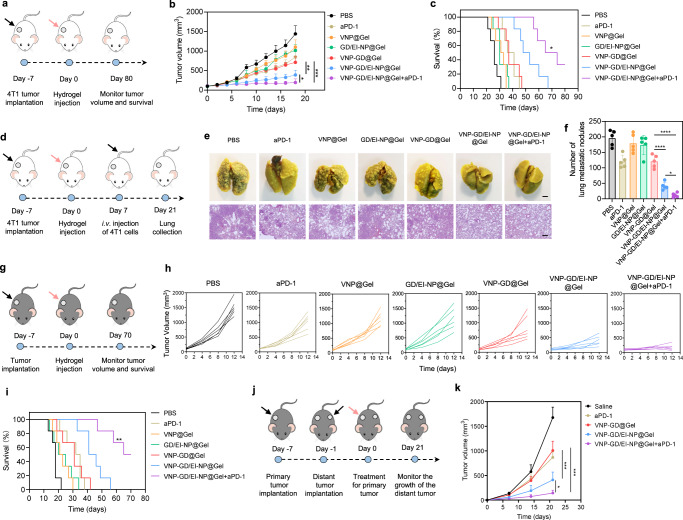
Antitumor efficacy in 4T1 metastatic triple-negative breast cancer models and B16F10 melanoma models. **a** Schematic illustration of the establishment and treatment strategy in 4T1 breast tumor model. **b** 4T1 tumor growth curves after different treatments. Data are presented as mean ± s.d. (*n* = 6 mice) and analyzed with two-way ANOVA followed by Tukey’s multiple comparisons test. VNP-GD/EI-NP@Gel+aPD-1 vs. VNP-GD/EI-NP@Gel: *P = 0.0259; VNP-GD/EI-NP@Gel vs. VNP-GD@Gel: **P = 0.0046; VNP-GD@Gel vs. VNP-GD/EI-NP@Gel+aPD-1: ***P = 0.0001. **c** Survival curve of the 4T1 tumor-bearing mice after different treatments (*n* = 6 mice). Data are analyzed with Log-rank (Mantel-Cox) test. VNP-GD/EI-NP@Gel vs. VNP-GD@Gel: **P = 0.0085; VNP-GD/EI-NP@Gel+aPD-1 vs. VNP-GD@Gel: ***P = 0.0005; VNP-GD/EI-NP@Gel+aPD-1 vs. VNP-GD/EI-NP@Gel: *P = 0.0308. **d** Schematic illustration of the establishment and treatment strategy of the breast tumor lung metastasis model. **e** Representative lung images and H&E assay after different treatments (*n* = 5 mice, scale bar = 2 mm for lung tissue images and 200 µm for H&E staining images). **f** Number of surface lung metastatic nodules in different treatment groups. Data are presented as mean ± s.d. (*n* = 5 mice) and analyzed with one-way ANOVA followed by Tukey’s multiple comparisons test. VNP-GD/EI-NP@Gel vs. VNP-GD@Gel: ****P < 0.0001; VNP-GD/EI-NP@Gel+aPD-1 vs. VNP-GD/EI-NP@Gel: *P = 0.0478. **g** Schematic illustration of the establishment and treatment strategy in B16F10 melanoma model. **h** B16F10 tumor growth curves after the same treatment regimen described above (n = 6 mice). **i** Survival curve of the B16F10 tumor-bearing mice after the same treatment regimen described above (*n* = 6 mice). Data are analyzed with Log-rank (Mantel-Cox) test (*n* = 6 mice). VNP-GD/EI-NP@Gel vs. VNP-GD@Gel: *P = 0.0173^;^ VNP-GD/EI-NP@Gel+aPD-1 vs. VNP-GD@Gel: ***P = 0.0005; VNP-GD/EI-NP@Gel+aPD-1 vs. VNP-GD/EI-NP@Gel: **P = 0.0031. **j** Schematic illustration of the establishment and treatment strategy of the B16F10 double tumor model. **k** Distant tumor growth curves after different treatments as described above. Data are presented as mean ± s.d. (*n* = 6 mice) and analyzed with two-way ANOVA followed by Tukey’s multiple comparisons test. VNP-GD/EI-NP@Gel vs. VNP-GD@Gel: ***P = 0.0004; VNP-GD/EI-NP@Gel+aPD-1 vs. VNP-GD/EI-NP@Gel: *P = 0.0187; VNP-GD@Gel vs. VNP-GD/EI-NP@Gel+aPD-1: ***P = 0.0001. Source data are provided as a Source Data file.

The in vivo pyroptosis-triggered tumor immunotherapy efficacy of VNP-GD and EI-NP was first evaluated on a 4T1 breast cancer model (Fig. 4a). To enable the in vivo delivery of VNP-GD and EI-NP, an injectable thermoresponsive Pluronic™ F-127-based hydrogel was developed for peritumoral administration. VNP-GD and EI-NP were co-loaded into the Pluronic F127 solution at room temperature, which formed in a hydrogel for about 1 min at 37 °C through a sol-gel transition (Supplementary Fig. 18). Additionally, the release of the bacteria from the hydrogel was evaluated, and more than 65% of the VNP-GD was released within 72 h (Supplementary Fig. 19). To further characterize the tumoral distribution of VNP-GD after peritumoral administration, GSDMD proteins were labeled with Rhodamine B for tumor tissue imaging. As shown in Supplementary Fig. 20, in the VNP-GD/EI-NP@Gel group, there were more Rhodamine B-labeled GD proteins distributed in the tumor than that in the GD/EI-NP@Gel group which could be attributed to the strong motility and hypoxia tropism of VNP improving the tumoral delivery and penetration of GD proteins.

As shown in Fig. 4b, VNP-GD/EI-NP@Gel (hydrogel co-loaded with VNP-GD and EI-NP) exhibited superior antitumor efficacy than VNP-GD@Gel and displayed the best tumor growth inhibition when combined with aPD-1 antibodies (VNP-GD/EI-NP@Gel+aPD-1 group). The average tumor volume in the VNP-GD/EI-NP@Gel+aPD-1 group on day 18 was 2- and 3.7-fold smaller than that in VNP-GD/EI-NP@Gel and VNP-GD@Gel groups, respectively. Additionally, the VNP-GD/EI-NP@Gel+aPD-1 prolonged the survival time of the 4T1 tumor-bearing mice with a median survival of 69.5 days, which was significantly longer than that of VNP-GD/EI-NP@Gel (50.5 days), indicating that the immune checkpoint blockade could synergize with the pyroptosis-mediated immune response to improve antitumor immunotherapy efficacy (Fig. 4c). Notably, compared with VNP-GD@Gel group (median survival, 37 days), VNP-GD/EI-NP@Gel treatment achieved better antitumor efficacy, demonstrating the importance of blocking ESCRT III-mediated cell membrane repair for enhancing tumor cell pyroptosis. Considering that lung metastasis is closely associated with breast cancer patients in the clinic and causes high mortality^36,37^, we further applied VNP-GD/EI-NP@Gel+aPD-1 treatment strategy on a 4T1 breast cancer lung metastasis model. In an established 4T1 breast cancer lung metastasis model (Fig. 4d), it was demonstrated that VNP-GD/EI-NP@Gel+aPD-1 potently inhibited lung metastasis and decreased the number of lung metastatic nodules compared to both VNP-GD@Gel and VNP-GD/EI-NP@Gel groups, as evidenced by the lung images and H&E stainings (Fig. 4e, f). Notably, the number of surface lung metastasis nodules in the VNP-GD@Gel group was 2.9-fold higher than that in the VNP-GD/EI-NP@Gel group, suggesting that enhancing pyroptosis by blocking ESCRT-mediated cell membrane repair holds great potential to prevent lung metastasis.

Moreover, to validate the broad applicability of the VNP-GD/EI-NP@Gel+aPD-1 treatment strategy, a B16F10 melanoma tumor model was established and treated with various formulations (Fig. 4g). As shown in Fig. 4h, the B16F10 tumors in the PBS control group displayed a sharp increase in the tumor volumes with all over 1000 mm^3^ on day 12, while both the VNP@Gel and GD/EI-NP@Gel showed the slight inhibition of the tumor growth. Notably, the tumor growth in the VNP-GD/EI-NP@Gel group was much slower than that in the VNP-GD@Gel group, which highlighted the importance of EI-NP in improving tumor pyroptosis-induced antitumor immune response. The best treatment efficacy of VNP-GD/EI-NP@Gel+aPD-1 was further substantiated, as evidenced by the smallest tumor volumes among all the treatment groups (Fig. 4h). Moreover, VNP-GD/EI-NP@Gel+aPD-1 prolonged the median survival of the tumor-bearing mice from 18 days (PBS) to 67.5 days, while VNP@Gel and GD/EI-NP@Gel only prolonged the median survival to 22 days and 22.5 days respectively. Notably, the more prolonged median survival of VNP-GD/EI-NP@Gel (43.5 days) compared with VNP-GD@Gel (31 days) further demonstrated that blocking ESCRT-mediated cell membrane repair could enhance tumor pyroptosis-mediated antitumor efficacy (Fig. 4i). Furthermore, to investigate if this local treatment could induce a systemic immune response, a distant tumor model was established as illustrated in Fig. 4j. The VNP-GD/EI-NP@Gel treatment showed a better tumor inhibition than the VNP-GD@Gel treatment group indicating a stronger systemic immune activation induced by the pyroptosis-mediated tumor antigen release that resulted from the combination of GSDMD-induced and ESCRT III inhibition-boosted tumor cell pyroptosis. In addition, the distant tumor volumes in the VNP-GD/EI-NP@Gel+aPD-1 group were significantly smaller than that in the VNP-GD/EI-NP@Gel group, demonstrating the synergistic efficacy when combined with aPD-1 antibodies to block PD-1/PD-L1 pathway to re-activate T cells for antitumor immunity (Fig. 4k). To enhance the biosafety, we utilized a hydrogel reservoir to realize local delivery of a safe dosage of VNP20009 to the tumor tissue. Leveraging the hypoxia targeting ability of the intracellular facultative anaerobic bacteria VNP20009, the delivery strategy would achieve the maximum antitumor effect in tumors while minimizing systemic toxicity and side effects. Moreover, both in the 4T1 tumor model and B16F10 tumor model, the body weight of the mice showed no significant decrease after the treatment of the hydrogel-based delivery systems (Supplementary Figs. 21, 22). In addition, H&E assay of the major organs in the C57BL6 mice further demonstrated a good biosafety profile of the hydrogel-based delivery system (Supplementary Fig. 23). Furthermore, according to the complete blood cell counts and systemic cytokine ELISA assay, hydrogel-based VNP-GD local delivery strategy did not induce systematic inflammation and related toxicity (Supplementary Figs. 24, 25).

### Preventing ESCRT-dependent cell membrane repair enhanced pyroptosis and augmented antitumor immune response

**Fig. 5:**
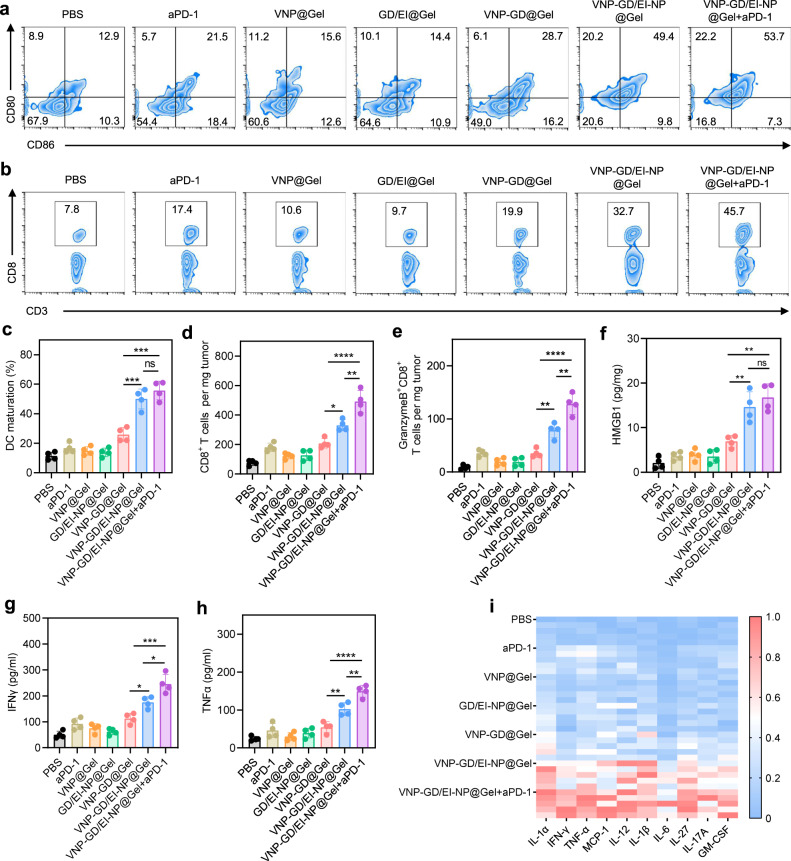
Preventing ESCRT-dependent cell membrane repair enhanced pyroptosis and augmented antitumor immune response. **a** Flow cytometry assay of dendritic cell maturation in the lymph nodes after treatments with PBS, aPD-1, VNP@Gel, GD/EI-NP@Gel, VNP-GD@Gel, VNP-GD/EI-NP@Gel and VNP-GD/EI-NP@Gel+aPD-1 (*n* = 4 mice). **b** Flow cytometry assay of CD8^+^ T cell infiltration in the tumor tissue after treatments as mentioned above, *n* = 4 mice. **c** Dendritic cell maturation in the lymph nodes after different treatments. Data are shown as mean ± s.d. and analyzed with one way ANOVA followed by Tukey’s multiple comparisons test, *n* = 4 mice (VNP-GD@Gel vs. VNP-GD/EI-NP@Gel: ***P = 0.0008; VNP-GD@Gel vs. VNP-GD/EI-NP@Gel+aPD-1: ***P = 0.0002; VNP-GD/EI-NP@Gel vs. VNP-GD/EI-NP@Gel+aPD-1: ^ns^*P* = 0.4116). **d** The number of CD8^+^ T cells per mg tumor tissue after different treatments. Data are shown as mean ± s.d. and analyzed with one way ANOVA followed by Tukey’s multiple comparisons test, *n* = 4 mice (VNP-GD@Gel vs. VNP-GD/EI-NP@Gel: *P = 0.0215; VNP-GD@Gel vs. VNP-GD/EI-NP@Gel+aPD-1: ****P < 0.0001; VNP-GD/EI-NP@Gel vs. VNP-GD/EI-NP@Gel+aPD-1: **P = 0.0042). **e** The number of Granzyme B^+^CD8^+^ T cells per mg tumor tissue after different treatments, n = 4 mice (VNP-GD@Gel vs. VNP-GD/EI-NP@Gel: **P = 0.0065 VNP-GD@Gel vs. VNP-GD/EI-NP@Gel+aPD-1: ****P < 0.0001; VNP-GD/EI-NP@Gel vs. VNP-GD/EI-NP@Gel+aPD-1: **P = 0.0028). HMGB1 (**f**), IFNγ (**g**), and TNFα (**h**) expressions in the tumor tissues after different treatments detected by ELISA. Data are shown as mean ± s.d. and analyzed with one way ANOVA followed by Tukey’s multiple comparisons test, *n* = 4 mice. (f, VNP-GD@Gel vs. VNP-GD/EI-NP@Gel: **P = 0.0080; VNP-GD@Gel vs. VNP-GD/EI-NP@Gel+aPD-1: **P = 0.0017; VNP-GD/EI-N*P*@Gel vs. VNP-GD/EI-NP@Gel+aPD-1: ^ns^*P* = 0.5319. g, VNP-GD@Gel vs. VNP-GD/EI-NP@Gel: *P = 0.0256; VNP-GD@Gel vs. VNP-GD/EI-NP@Gel+aPD-1: ***P = 0.0002; VNP-GD/EI-N*P*@Gel vs. VNP-GD/EI-NP@Gel+aPD-1: *P = 0.0127. h, VNP-GD@Gel vs. VNP-GD/EI-NP@Gel: **P = 0.0047; VNP-GD@Gel vs. VNP-GD/EI-NP@Gel+aPD-1: ****P < 0.0001; VNP-GD/EI-N*P*@Gel vs. VNP-GD/EI-NP@Gel+aPD-1: **P = 0.0053.) **i** Luminex-based quantification of cytokines and chemokines, including IL-1α, IFN-γ, TNF-α, MCP-1, IL-12, IL-1β, IL-6, IL-27, IL-17A, GM-CSF (*n* = 5 mice). Source data are provided as a Source Data file.

Pyroptosis is a form of programmed cell death that produces a large number of inflammatory factors, releases tumor antigens, and initiates antigen-presenting cells (APC)-mediated adaptive immune responses^38^, which could be leveraged to reawaken the immune system and overcome the tumor immunosuppressive microenvironment. Therefore, the dendritic cell (DC) maturation was first investigated after different treatments, and it was found that VNP-GD@Gel increased the CD80^+^CD86^+^ proportion of DCs from 12.9% (PBS group) to 28.7%, while VNP-GD/EI-NP@Gel increased the DC maturation to 49.4%, significantly higher than that of VNP-GD@Gel group (Fig. 5a, c). The increased DC maturation in VNP-GD/EI-NP@Gel was probably attributed to the enhanced tumor antigen release after tumor cell pyroptosis mediated by the combination of tumor-selective intracellular delivery of GSDMD by VNP and membrane repair inhibition by EI-NP. Next, the CD8^+^ T cell infiltration at the tumor site was evaluated, and it was found that the VNP-GD/EI-NP@Gel+aPD-1 treatment increased the CD8^+^ T cell infiltration from 7.8% (PBS group) to 45.7%, significantly higher than that of the VNP-GD@Gel (19.9%) and VNP-GD/EI-NP@Gel (32.7%) **(**Fig. 5b, Supplementary Fig. 26). More specifically, the infiltrated CD8^+^ T cell number increased from ~76 per mg tumor (PBS group) to ~493 per mg tumor after the VNP-GD/EI-NP@Gel+aPD-1 treatment (Fig. 5d). Moreover, VNP-GD/EI-NP@Gel+aPD-1 also potently increased the number of activated Granzyme B^+^ CD8^+^ T cells in the tumor, which was significantly higher than that in the VNP-GD@Gel and VNP-GD/EI-NP@Gel groups (Fig. 5e).

Furthermore, according to the ELISA assay of HMGB1 expression in Fig. 5f, compared with the PBS group, VNP@Gel and GD/EI-NP@Gel slightly increased the HMGB1 expressions in the tumor tissues, while the VNP-GD@Gel displayed a threefold increase of HMGB1 expression. Notably, after blocking the ESCRT-based cell membrane repair through EI-NP, VNP-GD/EI-NP@Gel and VNP-GD/EI-NP@Gel+aPD-1 treatments showed 7.1- and 8.1-fold increases in HMGB1 expression, respectively, when compared to PBS group, which was also significantly higher than that of VNP-GD@Gel group. Moreover, as shown in Fig. 5g, VNP-GD@Gel treatment resulted in a 2.2-fold increase of IFNγ expression in the tumor tissue, while the VNP-GD/EI-NP@Gel treatment showed a 3.5-fold increase of IFNγ expression when compared to that in PBS group. Moreover, after combination with immune checkpoint blockade therapy, VNP-GD/EI-NP@Gel+aPD-1 treatment showed the highest IFNγ expression level among all treatment groups. Similarly, VNP-GD/EI-NP@Gel+aPD-1 also increased the TNFα expression, which was 1.5-fold higher than that of VNP-GD/EI-NP@Gel treatment and 2.7-fold higher than that of VNP-GD@Gel treatment (Fig. 5h). Furthermore, as shown in Fig. 5i, the potently elevated inflammatory cytokines and chemokines expression detected by LEGENDplex^TM^ multi-Analyte Flow assay kit after the VNP-GD/EI-NP@Gel+aPD-1 treatment further substantiated the strongest immune activation among all treatment groups.

### Lyophilized hydrogel-based cell patch for the treatment of inoperable ovarian cancer

**Fig. 6:**
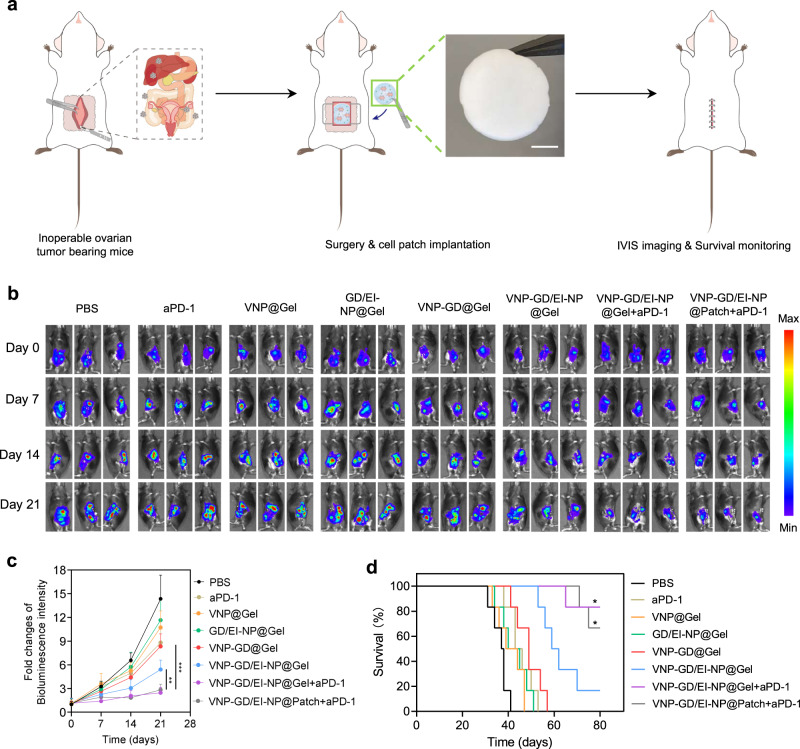
Antitumor efficacy of implantable cell patch in inoperable ovarian cancer. **a** Schematic illustration of the implantation of the cell patch for the treatment of inoperable ovarian cancer and the representative image of the lyophilized hydrogel patch loaded with EI-NP. Scale bar: 5 mm. **b** Representative bioluminescence images of the mice bearing ID8-Luc ovarian tumors after different treatments of PBS, aPD-1, VNP@Gel, GD/EI-NP@Gel, VNP-GD@Gel, VNP-GD/EI-NP@Gel, VNP-GD/EI-NP@Gel+aPD-1 and VNP-GD/EI-NP@Patch+aPD1 (*n* = 6 mice). **c** Data analysis of the normalized intensity of the bioluminescence signals of the mice with different treatments. Data are presented as mean ± s.d. (*n* = 6 mice) and analyzed with two-way ANOVA followed by Tukey’s multiple comparisons test. VNP-GD/EI-NP@Patch+aPD1 vs. VNP-GD/EI-NP@Gel: **P = 0.0078; VNP-GD/EI-NP@Patch+aPD1 vs. VNP-GD@Gel: ***P = 0.0005. **d** Survival curve of the ovarian tumor-bearing mice after different treatment of PBS, aPD-1, VNP@Gel (hydrogel loaded with VNP), GD/EI-NP@Gel (GSDMD protein cage, and EI-NP co-loaded in the hydrogel), VNP-GD@Gel (GSDMD protein cage-conjugated VNP loaded in the hydrogel), VNP-GD/EI-NP@Gel (GSDMD protein cage-conjugated VNP and EI-NP co-loaded in the hydrogel) and VNP-GD/EI-NP@Gel+aPD-1 (GSDMD protein cage-conjugated VNP and EI-NP co-loaded in the hydrogel with three times systemic injection of aPD-1 on day 0, day 2 and day 4) and VNP-GD/EI-NP@Patch+aPD1 (GSDMD protein cage-conjugated VNP and EI-NP loaded in the cell patch with three times systemic injection of aPD-1 on day 0, day 2 and day 4). Data are presented as mean ± s.d. (*n* = 6 mice) and analyzed by Log-rank (Mantel-Cox) test. VNP-GD/EI-NP@Patch+aPD1 vs. VNP-GD@Gel: *P = 0.0204. VNP-GD/EI-NP@Gel+aPD1 vs. VNP-GD@Gel: *P = 0.0169. Source data are provided as a Source Data file.

Ovarian cancer is a highly malignant tumor, and because of the lack of typical clinical symptoms in the early stage, many patients are associated with a large number of organ metastases in the abdominal cavity, including metastases in the liver, spleen, and kidney when diagnosed, which makes surgical treatment impossible^39,40^. Therefore, in view of this clinical challenge, for inoperable tumor treatment, such as advanced metastatic ovarian cancer, a pyroptosis-enhancing cell patch was prepared with a lyophilized hyaluronan hydrogel loaded with EI-NP (Supplementary Fig. 27). The lyophilized EI-NP showed great stability with negligible particle size change after 120 days (Supplementary Fig. 28), and the lyophilized hydrogel patch preloaded with EI-NP could serve as an off-the-shelf product for long-term storage. This patch could be easily implanted into the abdominal cavity after the addition of freshly prepared VNP-GD to treat metastatic ovarian cancer (Fig. 6a). As shown in Fig. 6b, c, the bioluminescence intensity from luciferase-expressing ID8 ovarian cancer cells in the VNP-GD/EI-NP@Gel group was significantly weaker than that in the VNP-GD@Gel group. Moreover, VNP-GD/EI-NP@Gel+aPD-1 and VNP-GD/EI-NP@Patch+aPD-1 showed similar antitumor efficacy, and both are much better than VNP-GD/EI-NP@Gel and VNP-GD@Gel treatments, suggesting the comparable and potent antitumor treatment efficacy of lyophilized cell patches. More importantly, VNP-GD/EI-NP@Gel prolonged the median survival of the inoperable ovarian tumor-bearing mice from 37.5 days (PBS group) to 60.5 days, which was significantly longer than that of VNP-GD@Gel group (49 days), demonstrating the importance of ESCRT inhibition in the pyroptosis-mediated cancer immunotherapy (Fig. 6d). VNP-GD/EI-NP@Gel+aPD-1 and VNP-GD/EI-NP@Patch+aPD-1 showed similar prolongation of the survival time, and over 65% of mice were still alive after 80 days indicating that the combination of immune checkpoint blockade with tumor pyroptosis could synergistically enhance antitumor immunity to improve treatment outcomes.

## Discussion

Pyroptosis, a highly inflammatory form of Gasdermin-dependent programmed cell death^41,42^, has recently attracted extensive attention from basic research to disease treatment. For instance, inhibiting immune cell pyroptosis can be utilized to treat inflammatory diseases such as cardiovascular disease and sepsis, while promoting cell pyroptosis can be used for antitumor immunotherapy^43–46^. However, for tumor cell pyroptosis, overexpression of DNA methyltransferase restricted the expression of Gasdermins, especially GSDME (DFNA5), which prevents the application of leveraging Gasdermin-dependent tumor cell pyroptosis for antitumor treatment^15^. To overcome this, the integration of DNA methyltransferase inhibitors that can increase the GSDME expression with chemical drugs that can activate the caspase-3 pathway to cleave GSDME for perforating membrane is trending now for triggering GSDME-mediated tumor pyroptosis^7,15,47^. Despite GSDME protein, other Gasdermin family proteins, including GSDMA, GSDMB, GSDMC, and GSDMD, have been found to trigger cell pyroptosis through an activated pore-forming domain unleashed by the distinct enzyme initiator^9^. Even though there are extensive mechanistic investigations of the Gasdermin family in triggering pyroptosis in immune cells and some cancer cells^2,48^, few have entered in vivo studies, particularly for antitumor applications. The reasons accounting for the lack of in vivo antitumor evaluations include the relatively restricted Gasdermin protein expression in tumor cells, the complication of initiating Gasdermin-mediated tumor cell pyroptosis requiring activation of multiple signaling pathways to unleash the pore-forming domain of Gasdermin proteins, and the presence of ESCRT III machinery-dependent membrane repair^9^. These augments work synergistically to inhibit the in vivo applications of Gasdermin-dependent pyroptosis. In this study, we presented a VNP bacteria-based GSDMD protein delivery system for in vivo antitumor treatment, in which VNP could efficiently shuttle GSDMD to the intracellular compartment of tumor cells where flagella on VNP-activated capapase-1 to further cleave delivered GSDMD to N-terminal domain for effective pore-forming and pyroptosis. To overcome ESCRT III machinery-induced cell membrane repair that can compromise the efficacy of tumor cell pyroptosis, a biodegradable nanoparticle was prepared to load Ca^2+^ chelator that can inhibit calcium influx to prevent the recruitment of ESCRT III machinery. Encouraging antitumor efficacy has been achieved in multiple tumor models through the combination of VNP-mediated delivery and activation of GSDMD proteins and EI-NP-induced inhibition of ESCRT III-dependent membrane repair.

Free GSDMD is hard to diffuse into tumor cells due to their relatively large molecular weight and negative surface charge, and the full length of GSDMD cannot trigger the cell pyroptosis due to the concealment of their active pore-forming domain^3,49^. To address this challenge, leveraging the superior tumor targeting ability of bacteria delivery systems^50^, we constructed a GSDMD protein cage (GD) with a GSH-responsive linker and further decorated GD on the surface of attenuated *Salmonella typhimurium* (VNP) to form VNP-GD. In this study, we demonstrated that GSDMD proteins could be efficiently transported to the cytoplasm of tumor cells, in which the VNP could trigger the activation of cleaved caspase 1 to transform intracellularly delivered GSDMD into pore-forming domain N-terminal GSDMD for binding to the plasma membrane and subsequently triggering pyroptosis in tumor cells. Furthermore, we found that ESCRT III-mediated membrane repair induced by the calcium influx during pyroptosis could help the tumor cells survive and increase the resistance of tumor cells to pyroptosis. Specifically, after the Gasdermin-NT-induced pore formation, calcium influx through the pore would trigger ESCRT-mediated membrane repair to subsequently bud and form macrovesicles, thus stopping the continuous expansion of cells and the release of inside antigens, diminishing the treatment efficacy of tumor pyroptosis. To address this issue, we engineered a biocompatible nanoparticle (EI-NP) to bioresponsively release Ca^2+^ chelator to inhibit calcium influx and subsequently prevent ESCRT-dependent membrane repair. We demonstrated that in vitro, the combination of VNP-GD and EI-NP could work synergistically to initiate and further strengthen the tumor cell pyroptosis, as evidenced by large ballooning bubbles formed and increased release of intracellular contents in both 4T1 and B16F10 cells. To enable in vivo antitumor applications, we developed two distinct formulations (an injectable hydrogel and a lyophilized cell patch) for treating multiple tumor settings. In the 4T1 and B16F10 tumor models, the VNP-GD/EI-NP@Gel elicited a strong local and systemic antitumor immune response that inhibits the primary and distant tumor growth and prevents the tumor metastasis. Further synergistic treatment efficacy was demonstrated when combining VNP-GD/EI-NP@Gel with systemic injection of immune checkpoint inhibitors. Enhanced tumor antigen release triggered by tumor pyroptosis can lead to more T cell infiltration and activation, which can be further strengthened by blocking PD-1/PD-L1 signaling pathway. To treat the clinically inoperable tumor such as late-stage ovarian cancer, we developed a VNP-GD/EI-NP@Gel cell path and implanted the patch into the abdominal cavity of mice, which significantly inhibited the tumor growth and spread and prolonged the survival time of the tumor-bearing mice.

Despite the superior properties of this pyroptosis-based antitumor strategy, there are still some limitations that need to be overcome for broader application and further clinical translation. First, the living bacteria VNP20009 have demonstrated a good biosafety profile in clinical trials, while at higher doses, there is still a risk of causing systemic toxicity. Thus, the long-term toxicity evaluation should be thoroughly performed. In addition, although this is an interlocking dedicated designed system, the preparation process of this pyroptosis strategy is relatively complex, and the storage and viability maintenance of cell products are also challenging. Therefore, future research efforts should be devoted to further optimizing the manufacturing conditions with simplified fabrication processes for future scale-up production.

In summary, we developed a hydrogel-based delivery system serving as a local reservoir to sustainedly release VNP-GD and EI-NP for enhanced programmed tumor cell death by integrating VNP-activated GSDMD-dependent tumor pyroptosis and inhibition of ESCRT III-mediated plasma membrane repair. This treatment strategy could further synergize with immune checkpoint blockade for improved immunotherapy efficacy against primary and metastatic tumors and inoperable tumors. Furthermore, the treatment strategy based on tumor pyroptosis has the potential to strengthen other immunotherapy modalities, such as adoptive T-cell therapies and cancer vaccines.

## Methods

### Cells and antibodies

The murine 4T1, B16F10 cell lines and VNP20009 were purchased from ATCC. Luciferase-expressed B16F10 and 4T1 were obtained from Imanis Life Sciences Inc. Luciferase-expressed ID8 cells were provided by Dr. Paula Hammond’s lab at MIT. Cells were cultured in the CO_2_ incubator (Fisher) at 37 °C with 5% CO_2_ and 90% relative humidity. The antibodies used in this study were summarized as follows (company, clone, category number): GoInVivo Purified anti-mouse CD279 (PD-1) (BioLegend, RMP1-14, 114114), Fluorescein isothiocyanate (FITC)-anti-mouse CD45 (BioLegend, 30-F11, 103108), APC-anti-mouse CD3 (BioLegend, 17A2, 100236), FITC-anti-mouse CD4 (BioLegend, GK1.5, 100406), PE-anti-mouse CD8a (BioLegend, 53-6.7, 100708), FITC-anti-mouse IFN*γ* (BioLegend, XMG1.2, 505806), PerCP/Cy5.5-anti-human/mouse Granzyme B (BioLegend, QA16A02, 372212), PE-anti-mouse CD45 (BioLegend, 30-F11, 103106), Alexa Fluro 594 anti-mouse CD8a (BioLegend, 53-6.7, 100758), FITC-anti-mouse CD11c (BioLegend, N418, 117306), PE-anti-mouse CD80 (BioLegend, 16-10A1, 104708), APC-anti-mouse CD86 (BioLegend, GL-1, 105012), Precision Count Beads (BioLegend, 424902). Mouse Reactive Pyroptosis Antibody Sampler Kit (Cell Signaling Technology, 98303 T), HRP Anti-beta Actin antibody (Abcam, ab49900). All antibody dilutions were performed following the manufacturer’s guidance (for flow cytometry assay: diluted by ~200 times for use; for western blot assay: antibodies from Cell Signaling Technology diluted by ~1000 times for use; HRP Anti-beta Actin antibody from Abcam: diluted by ~10,000 times for use).

### Synthesis of the GSH responsive linker

2-hydroxyethyl disulfide (R, 200 mg, 1.30 mmol) was dissolved in the anhydrous acetonitrile (ACN, 12 mL). *N*, *N*’-Disuccinimidyl carbonate (DSC, 1.33 g, 5.19 mmol) and Et_3_N (1.05 ml, 7.79 mmol) were added. The mixture was stirred for 8 h at room temperature under nitrogen protection, followed by the removal of the solvent under vacuum. The crude product was then dissolved in CH_2_Cl_2_ (20 mL). The solution was washed with saturated NaHCO_3_ solution, saturated NH_4_Cl solution, brine in sequence, and dried over anhydrous Na_2_SO_4_. The solvent in the organic phase was evaporated, and the solid that remained was recrystallized with ethyl acetate (20 ml). The resulting white solid was dried under vacuum (RS, 380 mg, yield 65%). ^1^H-NMR (CDCl_3_, 300 MHz): *δ* (ppm) 4.60 (t, 4H), 3.07 (t, 4H), 2.86 (s, 8H). ESI (m/z): calcd for C_14_H_16_N_2_O_10_S_2_, 436.4 [M]; found, 459.0 [M + Na]+.

### Synthesis, preparation, and characterization of ESCRT inhibitor-loaded dextran nanoparticles

Dextran (1.0 g, Mn ∼9–11 kDa) dissolved in 10 ml was added to a flame-dried round-bottom flask, then Pyridinium P-toluenesulfonate (15.6 mg, 0.062 mmol) and 2-ethoxypropene (4.16 mL, 37 mmol) were added during stirring. The reaction was stirred at room temperature for 30 min and quenched by adding 1 mL of triethylamine. The precipitated mixture was washed three times in basic water (pH ∼ 8) to prevent undesired degradation, centrifugated (7200 *g*, 15 min), and lyophilized to obtain the final white solid powder (m-dextran). To prepare the ESCRT inhibitor BAPTA-AM-loaded dextran nanoparticle, 10 mg m-dextran and 0.5 mg BAPTA-AM were dissolved in 2 mL dichloromethane. Then, 4 mL 3% poly (vinyl alcohol) (PVA) solution was added, and sonication (2 min in total, 2 s on, 2 s off, 40% power, ice bath) was performed. Next, the mixture was dispersed in 20 ml 0.3% PVA solution in the beaker and stirred for 1 h. The emulsion was centrifuged at 21,900 *g* for 35 min to collect the dextran nanoparticles (EI-NP). The supernatant was removed, and EI-NP was dispersed in 1 ml PBS. The particle size and zeta potential were measured with the Malvern Zetasizer instrument and analyzed with Malvern Zetasizer software. The morphology of EI-NP was observed under Transmission Electron Microscope (TEM, FEI Tecnai T-12 Cryo TEM system) imaging. To investigate the release profile of the ESCRT inhibitor, the EI-NP suspended in 2 ml PBS with 0.1% Tween 80 was loaded in a 20,000 MWCO dialysis cassette (Thermo scientific) for the analysis of drug release by high-performance liquid chromatography (HPLC, Agilent 1220 Infinity system) at different time points.

### Preparation and characterization of the protein cage-conjugated bacteria

To prepare the protein cages, the GSDMD proteins (MyBioSource, 0.0143 µmol) were dissolved in the PBS solution, then the GSH responsive linkers (0.214 µmol) dissolved in the DMSO were added and stirred at room temperature for 35 min to form the protein cage. The particle size and zeta potential of the protein cage were measured by the Malvern Zetasizer instrument. The morphology of the protein cages was observed under TEM imaging. Next, for the conjugation of protein cages on the surface of bacteria, the obtained protein cages and EDC/NHS (0.214 µmol) were added into the VNP20009 suspension (10^8^ CFU ml^−1^) and incubated for one hour in a 37 °C shaker incubator. The reaction mixture was centrifuged (1800 g, 5 min) to obtain the protein cage-conjugated bacteria (VNP-GD). The morphology of VNP-GD was observed under TEM imaging (FEI Tecnai T-12 Cryo TEM system). The surface conjugation of GD on VNP was verified by the western blot assay utilizing the specific anti-Gasdermin D antibody.

To verify the successful conjugation of the protein cage on the surface of the bacteria, the protein was labeled with Rhodamine B and formed into protein cages before being conjugated onto the bacteria. Centrifugation (1800 *g*) was performed to remove the unconjugated protein in the supernatant. Then the bacteria were labeled with Hoechst 33342 for 15 min at room temperature and washed with PBS three times before confocal imaging. The release of proteins from the protein cage-conjugated bacteria with or without the trigger of GSH (10 mM) was further characterized by measuring the protein concentration in the supernatant after centrifugation (1800 *g*) at different time points. Moreover, western blot assay utilizing specific anti-Gasdermin D antibody was performed to verify that the release proteins were Gasdermin D proteins.

### Characterization and verification of VNP-GD-induced tumor cell pyroptosis

2 × 10^5^ 4T1 cells were seeded into the confocal dishes, incubated overnight, and then treated with PBS, VNP, GD (GSDMD protein cage), VNP-GD (GSDMD protein cage-conjugated VNP), and VNP-GD+EI-NP (GSDMD protein cage-conjugated VNP+EI-NP) for 24 h (GSDMD = 2 μM, EI = 4 μM, VNP = 10^6^ CFU mL^−1^). After washing with PBS, the morphology of cells was observed under a confocal microscope. In addition, the Annexin V (staining at room temperature for 15 min) was used to label the cell membrane after pyroptosis. To further verify pyroptosis-mediated pore formation, 2 × 10^5^ 4T1 cells were seeded into the 24-well plate, incubated overnight, and then treated with PBS, VNP, GD, VNP-GD, and VNP-GD+EI-NP for 24 h (GSDMD = 2 μM, EI = 4 μM, VNP = 10^6^ CFU mL^−1^). SYTOX green and PI staining were performed respectively, and the cell uptake of the SYTOX green and PI were detected by the flow cytometry (Attune NxT Flow Cytometer System). Moreover, the LDH release was detected by the Invitrogen™ CyQUANT™ LDH Cytotoxicity Assay Kit according to the instruction and operation manual. HMGB1 expression was detected by the HMGB1 ELISA Kit accordingly and analysis with i-control 2.0 system.

### Western blot assay of the pyroptosis-related signaling pathway

5 × 10^5^ 4T1 cells were seeded into six-well plate, incubated overnight, and then treated with PBS, VNP, GD, VNP-GD, and VNP-G+EI-NP for 24 h (GSDMD = 2 μM, EI = 4 μM, VNP = 10^6^ CFU mL^−1^). Selective caspase 1 inhibitor (VRT-043198: 10 μM) and pan-caspase inhibitors (Q-VD-Oph: 50 μM; Ac-FLTD-CMK: 10 μM) were added to the parallel VNP-GD+EI-NP groups to verify the crucial role of caspases in the cleavage of GSDMD. After washing with PBS, the cells were digested with trypsin and collected into 1.5 ml tubes. Centrifugation (115 g, 4 min) was performed, and 100 μl Pierce^TM^ RIPA buffer with protease inhibitor cocktail was added to each tube for 1-h cell lysis with vortex every 10 min. Then, the lysed cells were centrifuged under 21,900 *g* for 30 min. The supernatant was collected, and BCA analysis was performed to determine the protein concentration. The protein samples added with protein loading buffer were boiled for 15 min at 100 °C water bath and stored at −20 °C for further use.

To perform the western blot assay, the 1× running buffer was prepared and gel plate was placed into the gel holder. Then the running buffer was added, and the protein marker and various samples were loaded accordingly. SDS-PAGE was performed at 120 V until the bromophenol blue indicator ran to the bottom. The proteins in the gels were further transferred onto a PVDF membrane (350 mA, 85 min). After incubation with 5% skim milk for 2 h at room temperature, the PVDF membranes were incubated with the primary rabbit antibodies (anti-Gasdermin D, anti-Cleaved Caspase 1, anti-Cleaved Gasdermin D, anti-HMGB1 respectively, dilution: 1:1000; anti-β-actin, dilution: 1:10000) at 4 °C overnight. The membranes were then washed with TBST buffer three times (each time 10 min) and incubated with the secondary antibody (Anti-rabbit IgG, HRP-linked, dilution: 1:1000) at room temperature for 1 h. After washing with TBST another three times, Western Blot Substrates were added to the membrane, and western blot images were taken by the iBright Imaging Systems.

### Flow cytometry assay of calcium influx and the confocal imaging of ESCRT III-mediated cell membrane repair during pyroptosis

For the flow cytometry assay of calcium influx, 2 × 10^5^ 4T1 cells were seeded into the 24-well plates, incubated overnight, and then treated with PBS, VNP, GD, VNP-GD, and VNP-GD+EI-NP for 24 h (GSDMD = 2 μM, EI = 4 μM, VNP = 10^6^ CFU mL^−1^). Cells were washed twice with PBS and incubated with 4 μM Fluo-8 for 45 min in the dark. Then cells were washed twice with PBS and digested with trypsin, and the fluorescence intensity of Fluo-8 was detected by the flow cytometry. For the confocal imaging of ESCRT III-mediated cell membrane repair, first, the plasmid containing the CHMP3-mCherry sequence was constructed by Addgene and extracted with the Monarch Plasmid Miniprep Kit according to the manufactureʼs instructions. The plasmid concentration was measured with a microplate reader, and the plasmids were stored at −20 °C for further use. To obtain 4T1 cells expressing CHMP3-mCherry, 2 × 10^5^ 4T1 cells were seeded into the 24-well plates and incubated overnight. Then, 2.5 μg CHMP3-Mcherry DNA plasmid and P 3000^TM^ Regent were mixed in 125 μl DEME medium without FBS in a 1.5 ml tube (A). Meantime, 7.5 μl Lipofectamine^TM^ 3000 was dissolved in 125 μl DEME medium without FBS in a 1.5 ml tube (B). Then the mixture in tube A was added to tube B and mixed well by pipetting and incubated for 10 min at room temperature. After washing with PBS, 600 μl DEME medium (with 10% FBS) was added to the 4T1 cell-containing confocal dishes. The final mixture in tube B was added slowly and evenly into the confocal dishes containing 4T1 cells. After 36 h, the expression of CHMP3-mCherry was verified by the confocal imaging. Then 4T1 cells expressing the CHMP3-mCherry were further treated with VNP-GD and VNP-GD+EI-NP for 24 h (GSDMD = 2 μM, EI = 4 μM, VNP = 10^6^ CFU mL^−1^). After washing with PBS, the cell membrane was labeled with Annexin V in binding buffer for 15 min under room temperature, and the confocal imaging and analysis (NIS-Elements Version 4.60) were performed to observe the ESCRT III-mediated cell membrane repair.

### Hydrogel preparation and bacteria release from the hydrogel

To prepare the Pluronic F127 thermosensitive hydrogel, 2.1 g Pluronic F127 was dissolved into 10 ml PBS solution under room temperature to form the 21% hydrogel solution. The gelation time of the 21% Pluronic F127 hydrogel was measured by incubating the hydrogel at 37 °C incubator, which formed into gel in 60–70 s. The injectable Pluronic F127 hydrogel delivery system was used for the treatment of 4T1 breast cancer model and B16F10 melanoma tumor model. To prepare the hyaluronic acid hydrogel, the Extralink®-Lite (PEGDA) was added to a mixture of Glycosil® (thiol-modified hyaluronan loaded with the bacteria delivery system) at 1:4 ratio, and it took about 30 min for the gelation. Then the bacteria release assay was performed after loading 10^7^ CFU bacteria into the hydrogel. The hydrogel loaded with bacteria was loaded in a cell strainer placed on a six-well plate, and then the six-well plate was submerged with PBS. At different time points, 100 µl of PBS solution in the plate was collected, diluted, and spread on LB medium plate with bacterial spreader. The number of released bacteria was calculated by counting the number of the formed clones accordingly. For the ID8-luc ovarian cancer treatment, the prepared EI-NP was loaded into hyaluronic acid hydrogel and lyophilization was performed to obtain the off-the-shelf hydrogel patch. VNP-GD was loaded into the hydrogel patch to form the final therapeutic cell patch before implantation. As for the stability assay, the particle size of EI-NP after lyophilization was measured at predetermined time points with the Malvern Zetasizer instrument.

### In vivo antitumor efficacy

The BALB/c (Female, aged 6-8 weeks) and C57BL/6 mice (Male and Female, aged 6-8 weeks) were purchased from Jackson laboratory. The animal study protocol was approved by the Institutional Animal Care and Use Committee at the University of Wisconsin-Madison. Mice were euthanized at humane endpoints if any of the following criteria were met: (i) weight loss or gain of >20%, (ii) moribund, (iii) severe abdominal swelling, (iv) jaundice, or (v) tumor volume >2000 mm^3^. To verify the antitumor efficacy of our treatment strategies, we first established the 4T1 breast cancer model by implanting 4T1 cells in the breast pad of the BALB/c mice. Seven days later, different formulations were loaded into the Pluronic F127 hydrogel for peritumoral injection, including PBS, aPD-1, VNP@Gel (hydrogel loaded with VNP), GD/EI-NP@Gel (GSDMD protein cage and EI-NP co-loaded in the hydrogel), VNP-GD@Gel (GSDMD protein cage-conjugated VNP loaded in the hydrogel), VNP-GD/EI-NP@Gel (GSDMD protein cage-conjugated VNP and EI-NP co-loaded in the hydrogel) and VNP-GD/EI-NP@Gel+aPD-1 (GSDMD protein cage-conjugated VNP and EI-NP co-loaded in the hydrogel with systemic injection of aPD-1 antibodies). GSDMD = 2 mg/kg, EI = 5 mg/kg, VNP = 10^7^ CFU per mouse, aPD-1 = 2.5 mg/kg (three doses on day 0, day 2 and day 4). The tumor volume was measured and calculated based on the equation: length × width^2^ × 0.5. The survival of the mice was monitored accordingly. To verify the biosafety profile of the therapeutic strategies, the body weight of the mice was monitored and the major organs were harvested for H&E assay. Moreover, the blood samples were collected to analyze the complete blood cell count with Abaxis HM5 Complete Blood Count (CBC) Analyzer and detect the cytokines levels with IL-6, IL-1β, and TNFα ELISA kits.

Next, a breast cancer lung metastasis model was established to evaluate the anti-metastasis effect of the hydrogel-based delivery systems. Briefly, 4T1 cells were injected into the breast pad of BALB/c mice on day −7, different treatments were administered by peritumoral injection after 7 days, including PBS, VNP@Gel, GD/EI-NP@Gel, VNP-GD@Gel, VNP-GD/EI-NP@Gel and VNP-GD/EI-NP@Gel+aPD-1. GSDMD = 2 mg/kg, EI = 5 mg/kg, VNP = 10^7^ CFU per mouse, aPD-1 = 2.5 mg/kg (three doses on day 0, day 2, and day 4). Then, on day 7, 2 × 10^5^ 4T1 cells were administered to the mice in different treatment groups by i.v. injection through tail vein. On day 21, the mice were euthanized, and the lungs were collected for further analysis. The lungs were washed with saline and fixed with Bouin’s solution for 6 h, and pictures were taken to observe the surface lung metastasis nodules. Furthermore, H&E assays were performed to observe the 4T1 tumor lung metastasis.

To further verify the antitumor efficacy of the hydrogel-based delivery systems, a melanoma tumor model was established by injecting B16F10 cells into the right flank of C57BL/6 mice on day −7. Seven days later, different formulations were loaded into the Pluronic F127 hydrogel for peritumoral injection, including PBS, aPD-1, VNP@Gel, GD/EI-NP@Gel, VNP-GD@Gel, VNP-GD/EI-NP@Gel and VNP-GD/EI-NP@Gel+aPD-1. GSDMD = 2 mg/kg, EI = 5 mg/kg, VNP = 10^7^ CFU per mouse, aPD-1 = 2.5 mg/kg (three doses on day 0, day 2 and day 4). The tumor volume was measured and calculated using the equation: length × width^2^ × 0.5. The survival of the mice was monitored accordingly. To verify if the local treatment strategy could activate the systemic immunity to inhibit the growth of the distant tumor, a double B16F10 tumor model was established. Briefly, on day −7, the primary B16F10 tumor was established by injecting the B16F10 cells into the right flank of the C57BL/6 mice. Six days later, the second tumor was established by injecting B16F10 cells on the left flank of the C57BL/6 mice. The tumor volume of the second tumor was monitored according to the equation: length × width^2^ × 0.5.

To extend our developed technology for treating inoperable tumors, an advanced ovarian tumor model was established. Briefly, 1 × 10^7^ ID8-Luc ovarian tumor cells were injected intraperitoneally into 6-week-old female C57BL/6 mice. One week later, the establishment of the ovarian tumor model was verified by the IVIS imaging system (PerkinElmer, version 4.5). D-luciferin potassium salt was dissolved into PBS and intraperitoneally injected into the mice (100 μl, 3 mg D-luciferin potassium salt per mouse). Five minutes after the injection, bioluminescence imaging was performed to record the distribution and growth of the ID8-Luc cells in the enterocoelia. After the establishment of the inoperable ovarian tumor model, surgery was performed to open the abdominal cavity of the mice, and different hydrogel-based delivery systems including PBS, aPD-1, VNP@Gel, GD/EI-NP@Gel, VNP-GD@Gel, VNP-GD/EI-NP@Gel, VNP-GD/EI-NP@Gel+aPD-1 and VNP-GD/EI-NP@Patch+aPD1 (GSDMD protein cage-conjugated VNP and EI-NP loaded in the cell patch with three times systemic injection of aPD-1) were implanted accordingly. GSDMD = 2 mg/kg, EI = 5 mg/kg, VNP = 10^7^ CFU per mouse, aPD-1 = 2.5 mg/kg (three doses on day 0, day 2 and day 4). IVIS imaging was performed at predetermined time points to monitor the tumor growth.

### In vivo immune activation

To verify the immune activation of our treatment strategies, we established the 4T1 breast cancer model by implanting 4T1 cells in the breast pad of the BALB/c mice. Seven days later, different formulations were loaded into the Pluronic F127 hydrogel for peritumoral injection, including PBS, aPD-1, VNP@Gel, GD/EI-NP@Gel, VNP-GD@Gel, VNP-GD/EI-NP@Gel, and VNP-GD/EI-NP@Gel+aPD-1. GSDMD = 2 mg/kg, EI = 5 mg/kg, VNP = 10^7^ CFU per mouse, aPD-1 = 2.5 mg/kg (three doses on day 0, day 2, and day 4). Then one week later, the tumors and lymph nodes were harvested, weighed, washed with PBS, cut into small pieces, and digested with DEME medium containing 0.5 mg/ml collagenase for 1 h at a 37 °C incubator. After the digested tumor tissues were mechanically disrupted, they were filtered through a 40 μm cell strainer. For the analysis of lymph nodes, the cell suspension was stained with anti-mouse CD11c, anti-mouse CD80, and anti-mouse CD86. For the analysis of tumor tissue, the cell suspension was stained with anti-mouse CD3, anti-mouse CD4, anti-mouse CD8a, and anti-mouse Granzyme B antibodies and analyzed using flow cytometry with Attune NxT Flow Cytometer software (All these antibodies were diluted by ~200 times). The cytokines expressions in the tumor tissue after different treatments were analyzed by the LEGENDplex^TM^ Multi-Analyte Flow Assay Kit, and IFNγ and TNFα ELISA kits according to the manufacture’s guidance.

### Statistics

All the results are shown as mean ± s.d.. The GraphPad Prism software was used to perform statistical analysis. Unpaired Student-*t* test was used to compare two groups and analysis of variance (ANOVA) was used to compare multiple groups (>two groups) statistically. Log-rank test was performed for the statistical analysis of the survival study. A *P* value lower than 0.05 (*P < 0.05) was considered the threshold for statistical significance among control groups and experimental groups.

### Reporting summary

Further information on research design is available in the Nature Research Reporting Summary linked to this article.

## Supplementary information

Supplementary Information

Reporting Summary

## Data Availability

The authors declare that all the data supporting the findings of this study are available within the article and Supplementary Information. Source data are provided with this paper.

## Source data

Source Data
