# Supplementary material for: Enhancing Gasdermin-induced tumor pyroptosis through preventing ESCRT-dependent cell membrane repair augments antitumor immune response
Source: Nat Commun. 2022 Oct 24;13:6321. doi: 10.1038/s41467-022-34036-8 (PMC9592600; doi:10.1038/s41467-022-34036-8)
Supplement: Supplementary file 1 — Supplementary Information [file 41467_2022_34036_MOESM1_ESM.pdf]

Enhancing Gasdermin-Induced Tumor Pyroptosis Through Preventing ESCRT-Dependent Cell Membrane Repair Augments Anti-Tumor Immune Response

Zhaoting Li[1,2,3], Fanyi Mo[1], Yixin Wang[1,2,3], Wen Li[1], Yu Chen[1,2,3], Jun Liu[1,2,3], Ting-Jing Chen-Mayfield[1], Quanyin Hu[1,2,3,*]

[1]Pharmaceutical Sciences Division, School of Pharmacy, University of Wisconsin-Madison, Madison, WI 53705, United States

[2]Carbone Cancer Center, School of Medicine and Public Health, University of Wisconsin-Madison, Madison, WI 53705, United States

[3]Wisconsin Center for NanoBioSystems, School of Pharmacy, University of Wisconsin-Madison, Madison, WI 53705, United States

Corresponding Author

*Quanyin Hu, Email: qhu66@wisc.edu

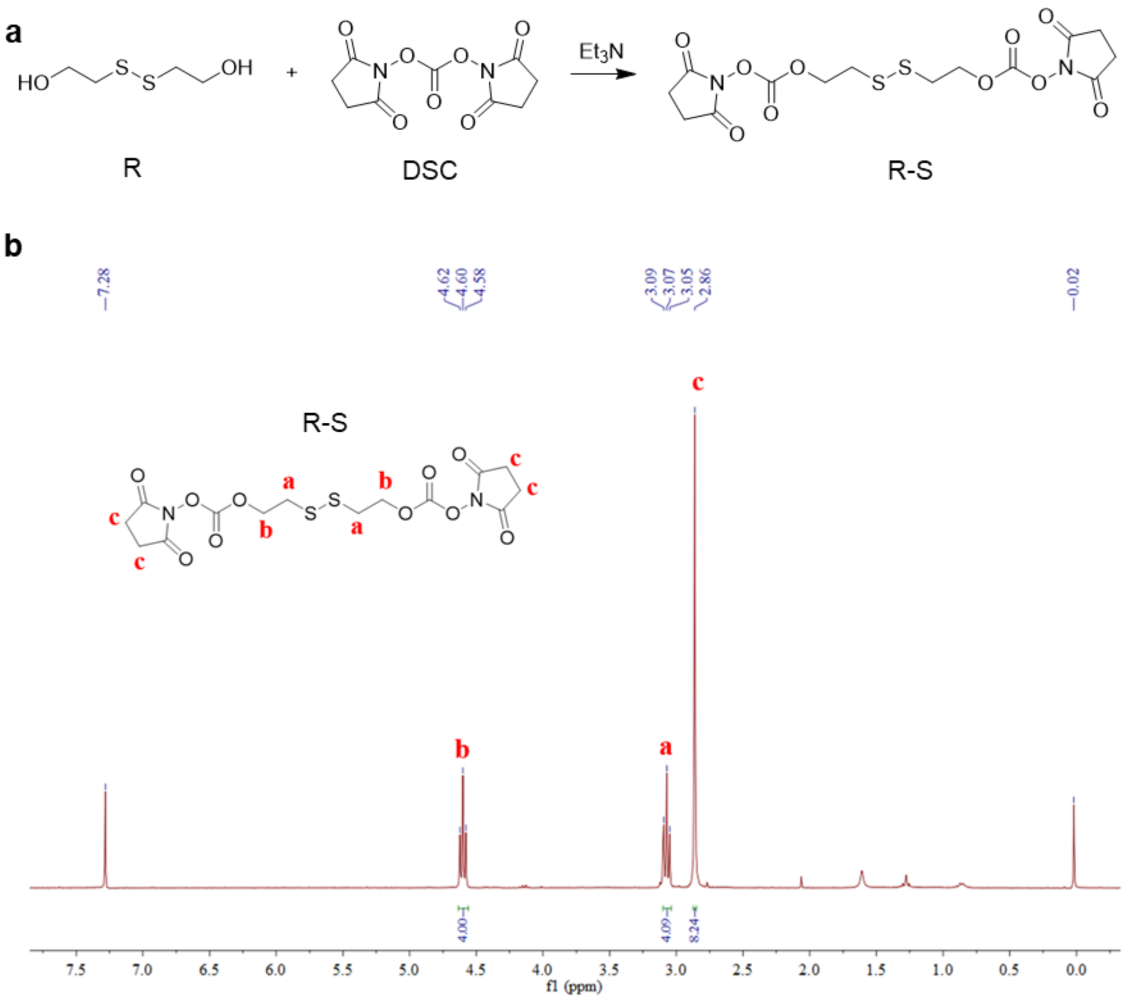

Supplementary Fig. 1 | Synthesis route and ^{1}H NMR characterization of the GSH responsive linker. a, Synthesis route of the GSH responsive linker (R-S). **b**, ^{1}H NMR characterization of the GSH responsive linker (R-S).

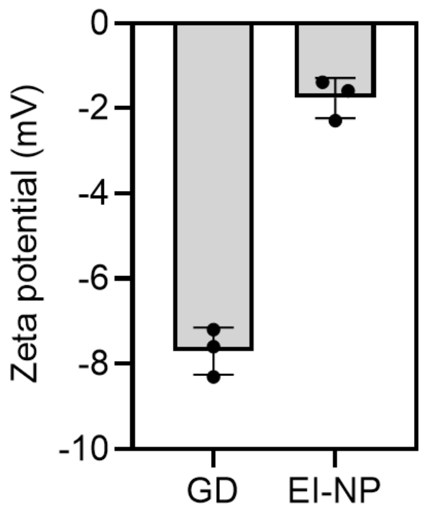

Supplementary Fig. 2 | Zeta potential of the Gasdermin protein cage GD and ESCRT inhibitor loaded nanoparticle EI-NP. Data are shown as mean ± s.d. (n = 3 biologically independent samples).

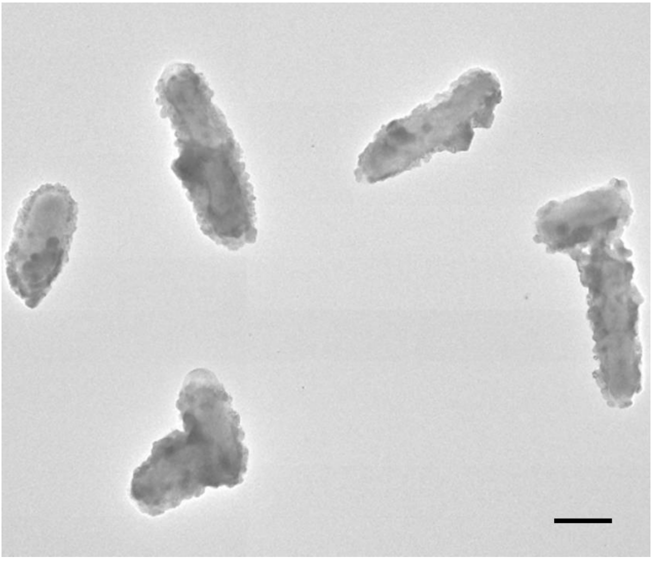

Supplementary Fig.3 | Representative TEM image of VNP-GD. Scale bar = 1 μm. The experiments were repeated three times independently.

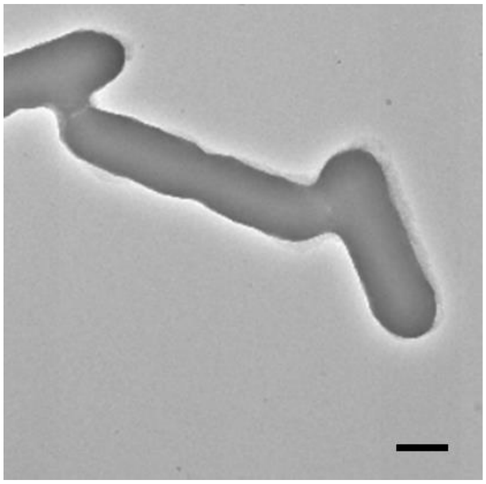

Supplementary Fig. 4 | Representative TEM image of VNP. Scale bar = 500 nm. The experiments were repeated three times independently.

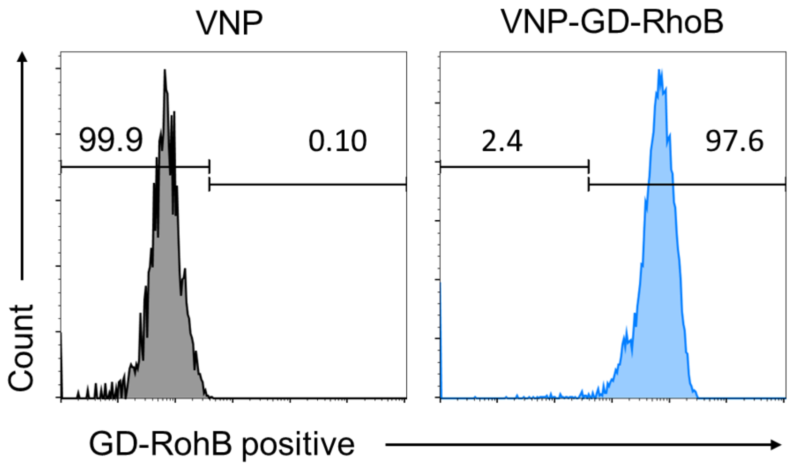

Supplementary Fig. 5 | Flow cytometry assay of the conjugation efficiency of Rhodamine B-labeled Gasdermin D protein cages on the surface of VNP (n = 3 biologically independent samples).

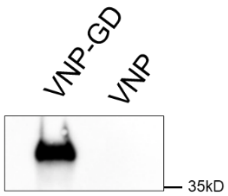

Supplementary Fig. 6 | Western blot assay of Gasdermin D in VNP-GD and VNP. The experiments were repeated three times independently.

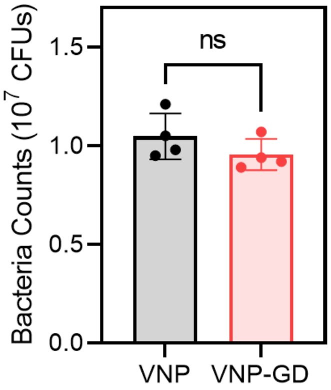

Supplementary Fig. 7 | Bacterial counts of VNP and VNP-GD after cultured on LB solid medium. Data are shown as mean ± s.d. (n = 4 biologically independent samples) and analyzed with two-tailed unpaired Student-*t* test.

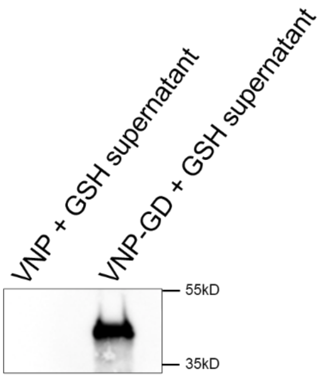

Supplementary Fig. 8 | Western blot verification of Gasdermin D releasing from VNP-GD after GSH (10 mM) treatment. The experiments were repeated three times independently.

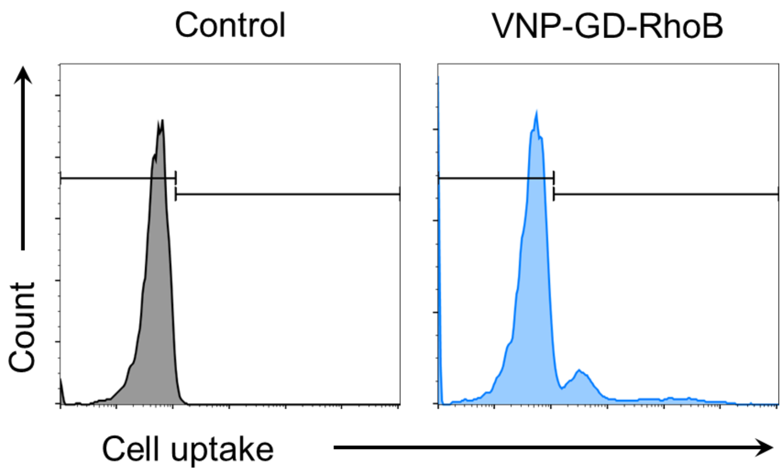

Supplementary Fig. 9 | Flow cytometry assay of the cell uptake of VNP-GD-RhoB in 4T1 cells (n = 3 biologically independent samples). The protein cages were labeled with NHS-Rhodamine B before conjugation onto the bacteria. Untreated 4T1 cells served as control.

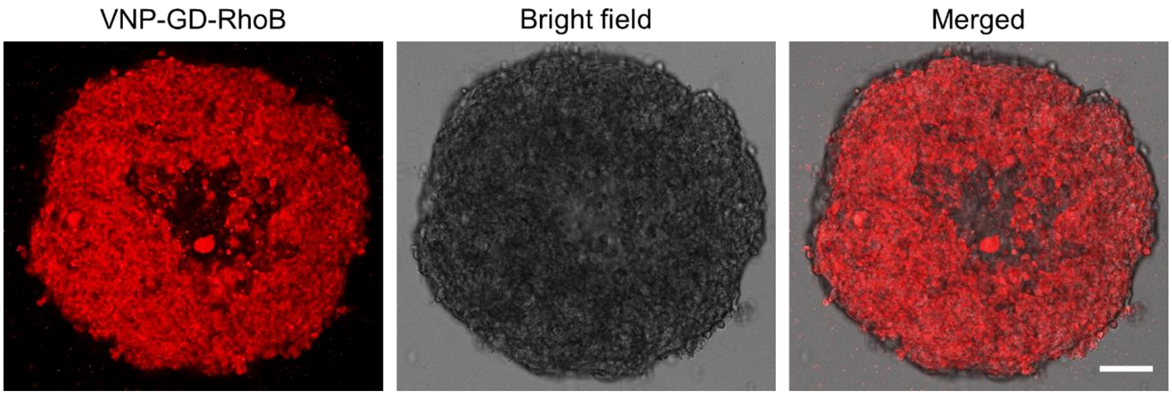

Supplementary Fig. 10 | Confocal images of penetration of VNP-GD. The protein cages were labeled with NHS-Rhodamine B before conjugation onto VNP. Scale bar = 100 μm. The experiments were repeated three times independently.

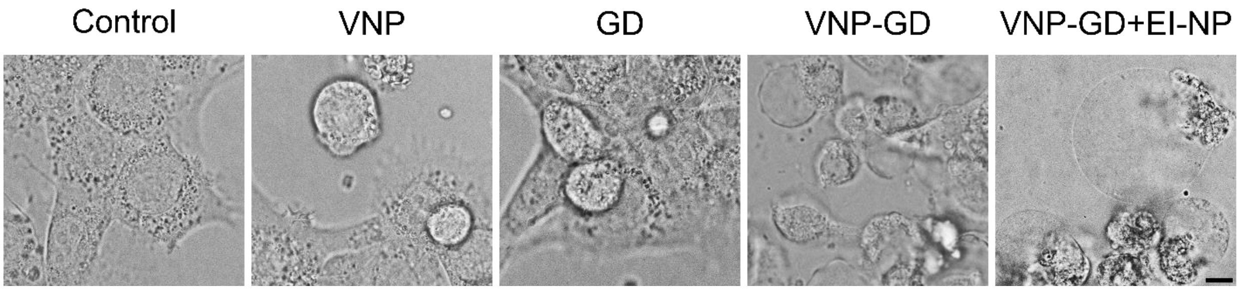

Supplementary Fig. 11 | Enlarged pictures of 4T1 cell pyroptosis in **Fig. 2a** after different treatments (scale bar = 5 μm). The experiments were repeated three times independently.

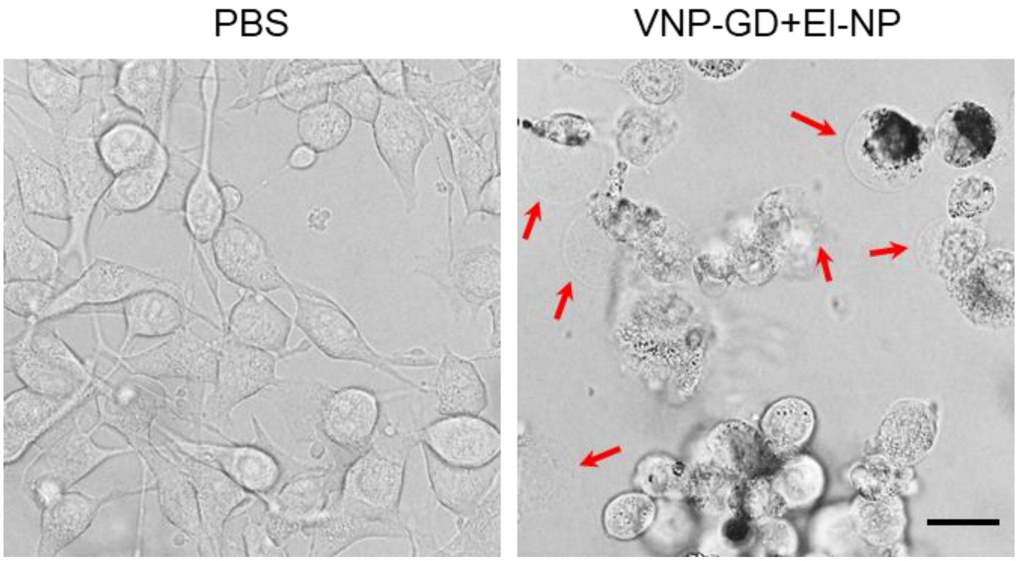

Supplementary Fig. 12 | Representative images of B16F10 undergoing pyroptosis after treatment of PBS and VNP-GD+EI-NP for 24 h. Scale bar = 20 μm. The experiments were repeated three times independently.

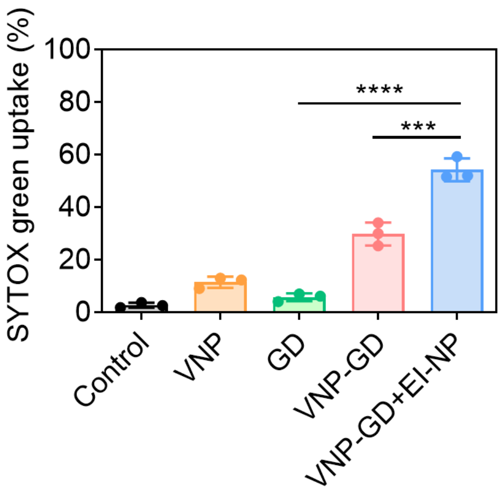

Supplementary Fig. 13 | Data analysis of the cell uptake of SYTOX green in 4T1 tumor cells after incubation with PBS, VNP, GD (GSDMD protein cage), VNP-GD (GSDMD protein cage-conjugated VNP), and VNP-GD+EI-NP (GSDMD protein cage-conjugated VNP + EI-NP) for 24 hours. Data are presented as mean ± s.d. (n = 3 biologically independent samples) and analyzed with one-way ANOVA followed by Dunnett's multiple comparisons test. VNP-GD+EI-NP vs. VNP-GD: $^{***}P = 0.0003$; VNP-GD+EI-NP vs. GD: $^{****}P < 0.0001$.

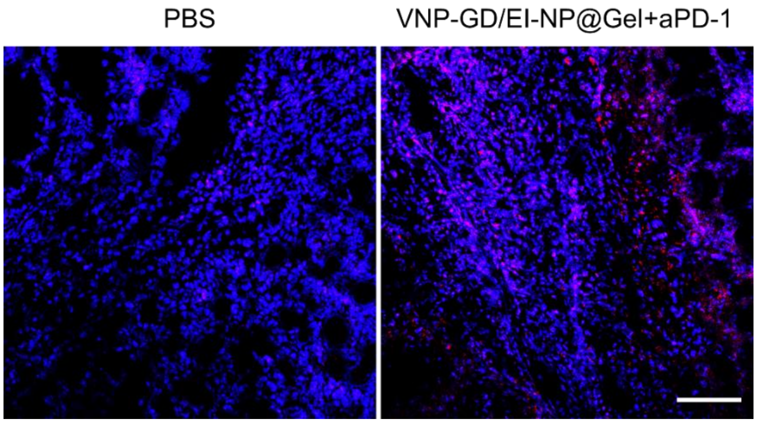

Supplementary Fig. 14 | Immunofluorescence assay of cleaved caspase-1 after PBS and VNP-GD/EI-NP@Gel+aPD-1 treatments (n = 3 mice, scale bar = 100 μm).

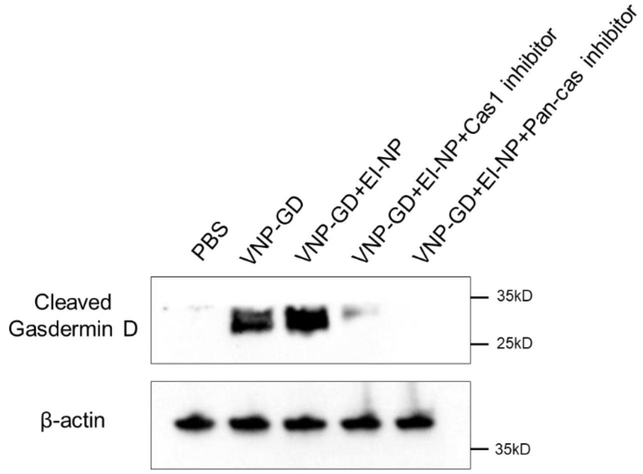

Supplementary Fig. 15 | Western blot assay of cleaved Gasdermin D after different treatments with caspase inhibitors. The experiments were repeated three times independently.

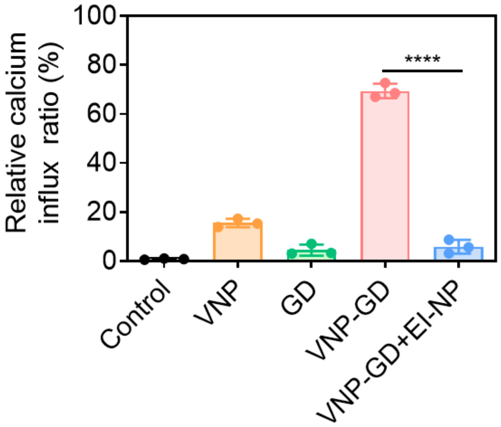

Supplementary Fig. 16 | Quantified relative calcium influx ratio. Data are presented as mean ± s.d. and analyzed with one-way ANOVA followed by Dunnett's multiple comparisons test ($^{****}P<0.0001$, n = 3 biologically independent samples).

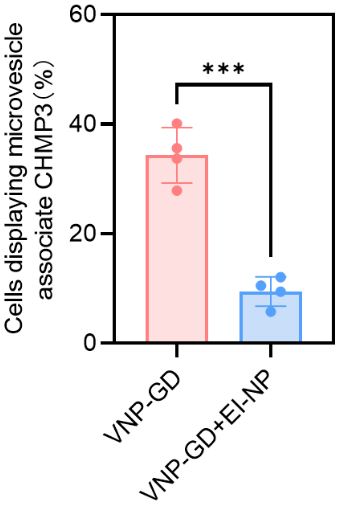

Supplementary Fig. 17 | Data analysis of the percentage of cells displaying microvesicle-associated CHMP3 in Fig. 3e (Data are presented as mean ± s.d. and analyzed with two-tailed unpaired Student-*t* test, $^{***}P= 0.0001$, n = 4 biologically independent samples).

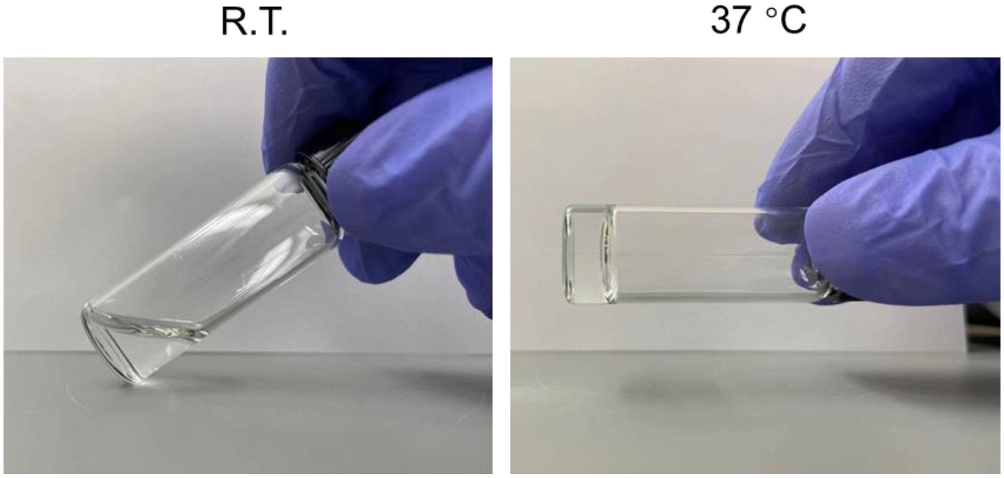

Supplementary Fig. 18 | Gel formation of the Pluronic® F-127 solution at room temperature and 37 °C.

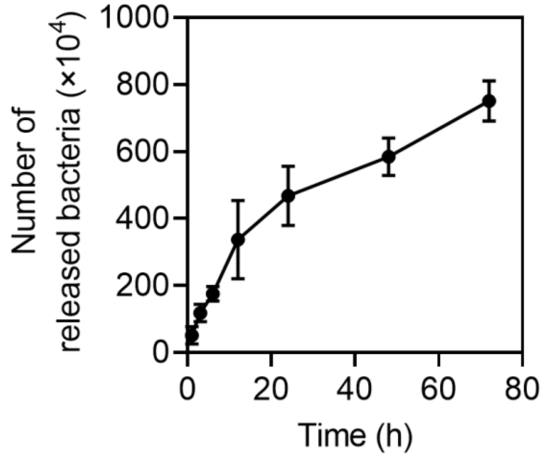

Supplementary Fig. 19 | Number of the bacteria released from hydrogel. 1×10^7 CFU bacteria were loaded in the hydrogel. Data are shown as mean ± s.d. (n = 3 biologically independent samples).

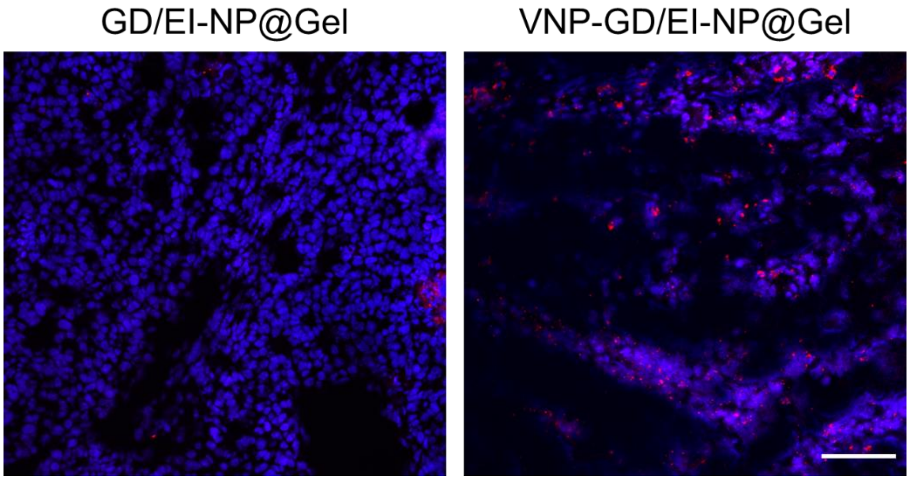

Supplementary Fig.20 | Representative confocal images of the distribution of Rhodamine B-labeled GSDMD in the tumor tissue after peritumoral injection of GD/EI-NP@Gel and VNP-GD/EI-NP@Gel (n = 3 mice). Scale bar = 100 μm. The experiments were repeated three times independently.

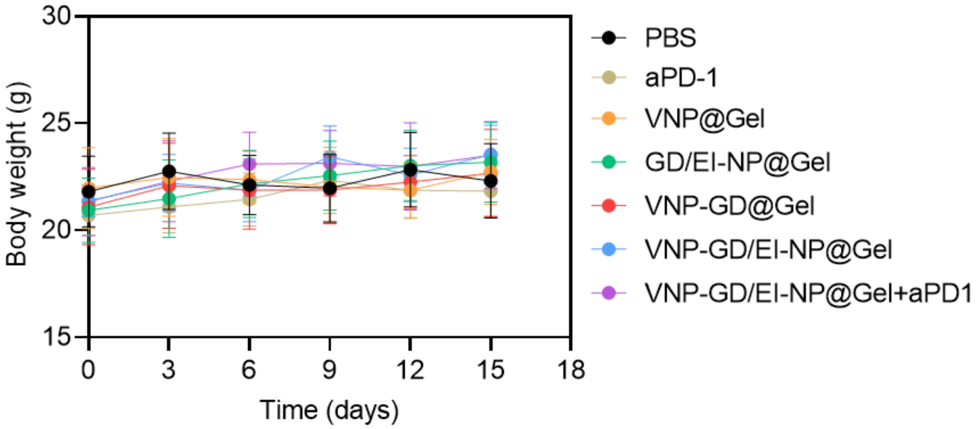

Supplementary Fig. 21 | Body weight changes of the 4T1 breast tumor-bearing mice after treatment with PBS, aPD-1, VNP@Gel (hydrogel loaded with VNP), GD/EI-NP@Gel (GSDMD protein cage and EI-NP co-loaded in the hydrogel), VNP-GD@Gel (GSDMD protein cage-armed VNP loaded in the hydrogel), VNP-GD/EI-NP@Gel (GSDMD protein cage-armed VNP and EI-NP co-loaded in the hydrogel) and VNP-GD/EI-NP@Gel+aPD-1 (GSDMD protein cage-armed VNP and EI-NP co-loaded in the hydrogel with systemic injection of aPD-1 antibodies). GSDMD = 2 mg/kg, EI = 5 mg/kg, VNP = 10^7 CFU per mouse, aPD-1 = 2.5 mg/kg (three doses on day 0, day 2 and day 4). Data are presented as mean ± s.d. (n = 6 mice).

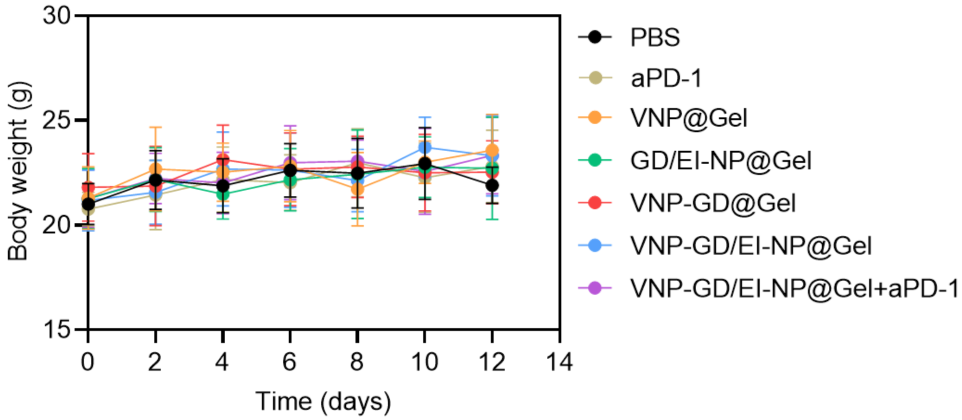

Supplementary Fig. 22 | Body weight changes of the B16F10 tumor-bearing mice after treatment with PBS, aPD-1, VNP@Gel (hydrogel loaded with VNP), GD/EI-NP@Gel (GSDMD protein cage and EI-NP co-loaded in the hydrogel), VNP-GD@Gel (GSDMD protein cage-armed VNP loaded in the hydrogel), VNP-GD/EI-NP@Gel (GSDMD protein cage-armed VNP and EI-NP co-loaded in the hydrogel) and VNP-GD/EI-NP@Gel+aPD-1 (GSDMD protein cage-armed VNP and EI-NP co-loaded in the hydrogel with systemic injection of aPD-1 antibodies). GSDMD = 2 mg/kg, EI = 5 mg/kg, VNP = 10^7 CFU per mouse, aPD-1 = 2.5 mg/kg (three doses on day 0, day 2 and day 4). Data are presented as mean ± s.d. (n = 6 mice).

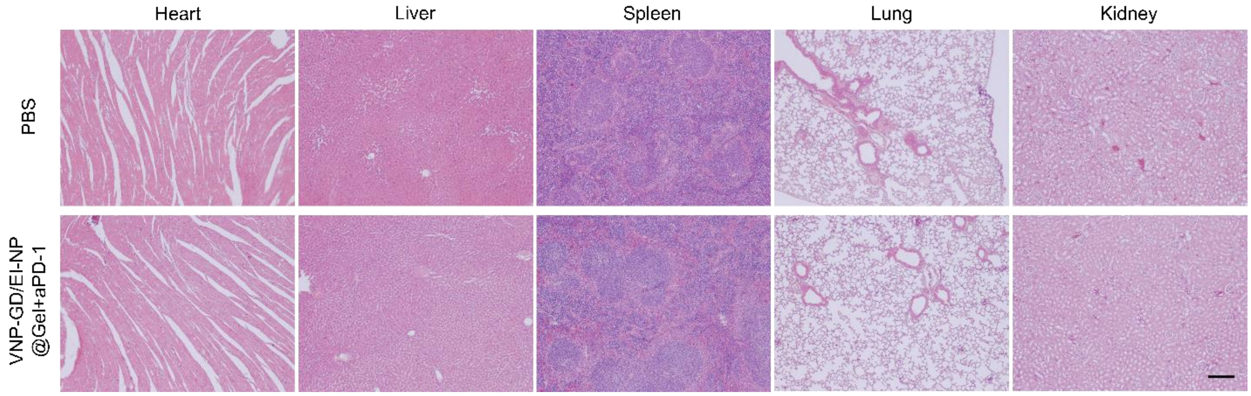

Supplementary Fig. 23 | H&E assay of different organs including heart, liver, spleen, lung and kidney of C57BL/6 mice after treatments with PBS and VNP-GD/EI-NP@Gel+aPD-1. GSDMD = 2 mg/kg, EI = 5 mg/kg, VNP = 10^7 CFU per mouse, aPD-1 = 2.5 mg/kg (three doses on day 0, day 2 and day 4) (n = 3 mice). Scale bar = 200 μm. The experiments were repeated three times independently.

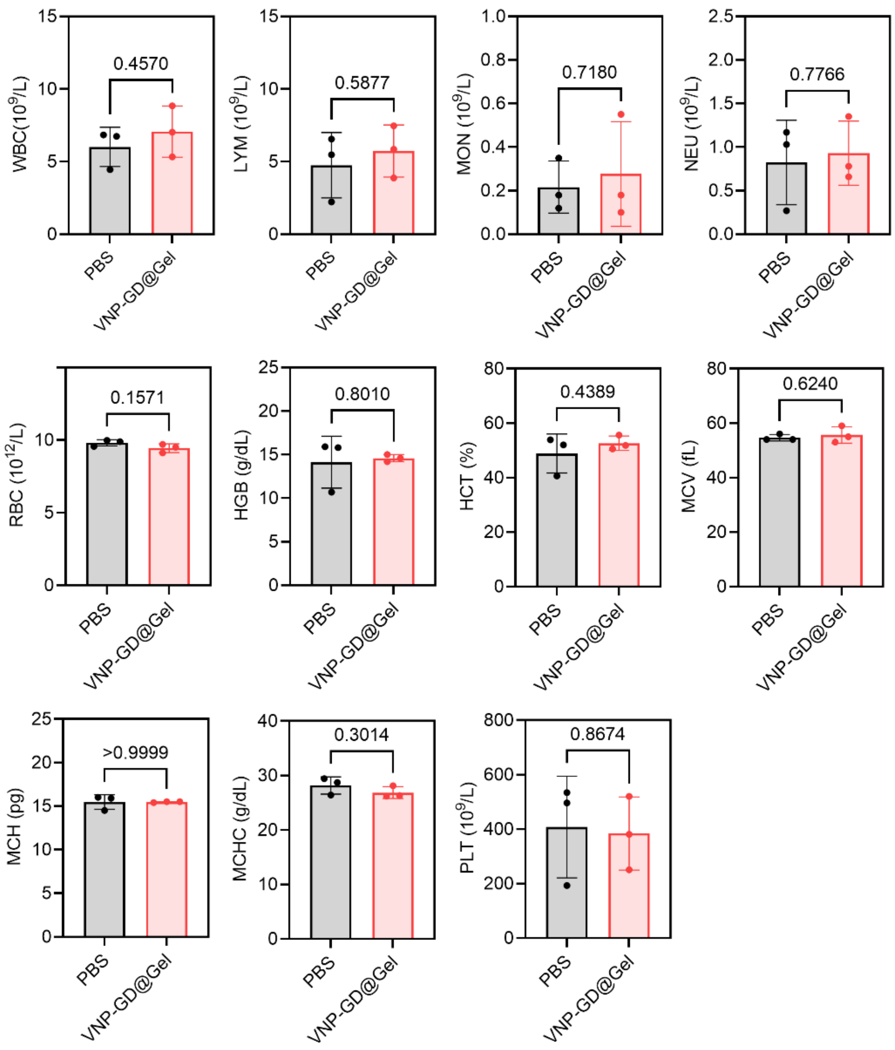

Supplementary Fig. 24 | Complete blood count analysis after PBS or VNP-GD@Gel treatments in Balb/c mice. Data are presented as mean ± s.d. and analyzed with two-tailed unpaired Student-*t* test (n = 3 mice). WBC: White Blood Cell Count; LYM: Lymphocytes; MON: Monocytes; NEU: Neutrophils; BAS: Basophils; RBC: Red Blood Cell Count; HGB: Hemoglobin; HCT: Hematocrit; MCV: Mean Corpuscular Volume; MCH: Mean Corpuscular Hemoglobin; MCHC: Mean Corpuscular Hemoglobin Concentration; PLT: Platelet Count.

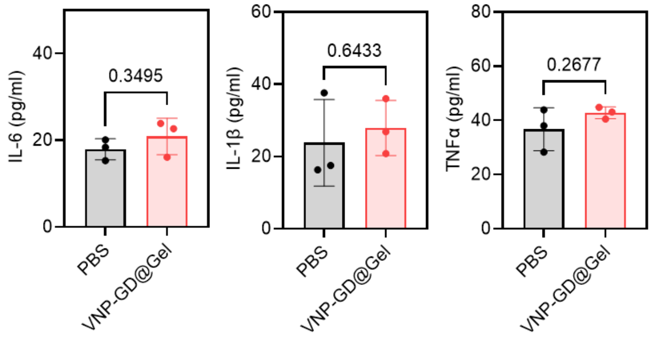

Supplementary Fig. 25 | Systemic cytokine detections after PBS or VNP-GD@Gel treatments in Balb/c mice. Data are presented as mean ± s.d. and analyzed with two-tailed unpaired Student-*t* test (n = 3 mice).

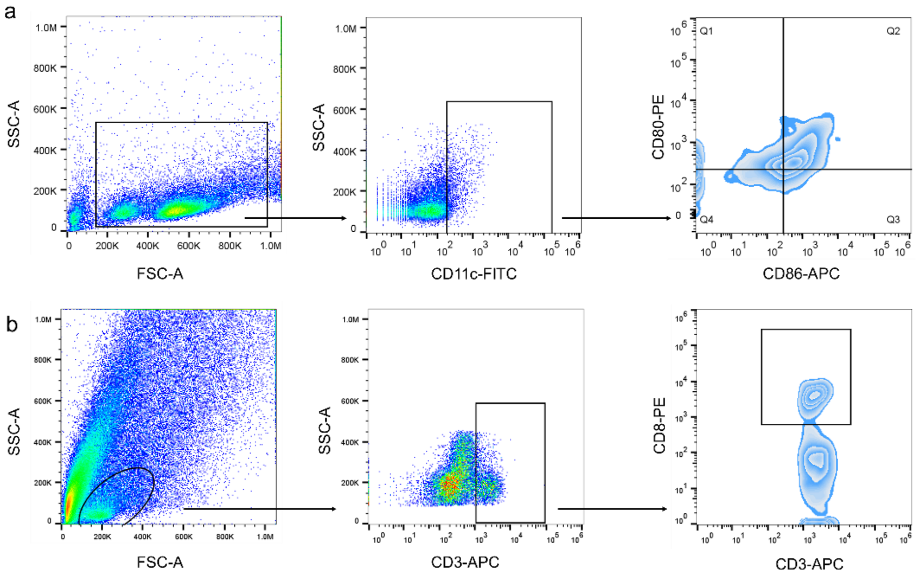

Supplementary Fig. 26 | Gating strategy for flow cytometry assay of dendritic cells in lymph nodes and CD8$^+$ T cell infiltration in the tumor tissue. **a**. Representative gating strategy for dendritic cell detection in Fig. 5a, 5c. **b**. Representative gating strategy for CD8$^+$ T cell detection in Fig. 5b, 5d, 5e.

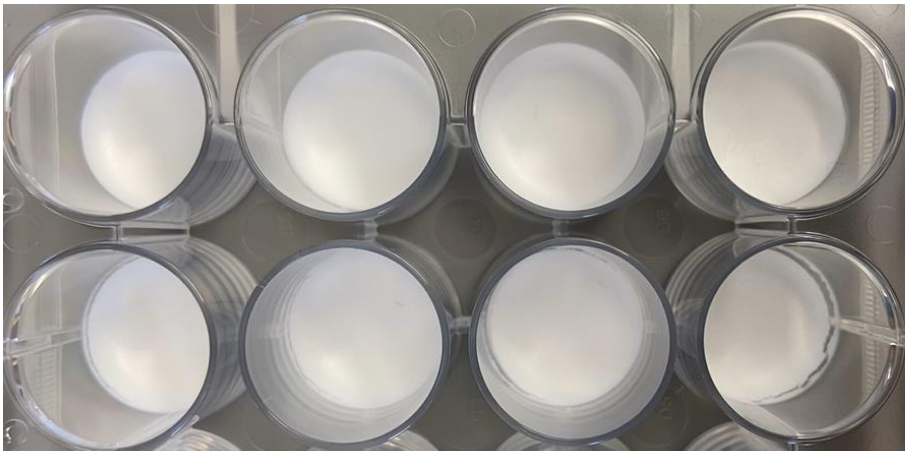

Supplementary Fig. 27 | Representative image of lyophilized hydrogel patches loaded with EI-NP.

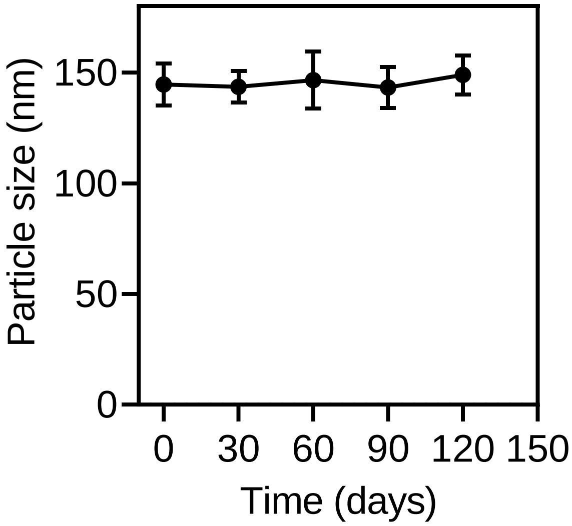

Supplementary Fig. 28 | Stability of re-suspended dextran nanoparticle (EI-NP) after lyophilization. Data are shown as mean ± s.d. (n = 3 biologically independent samples).
