# Supplementary material for: Enhancing Gasdermin-induced tumor pyroptosis through preventing ESCRT-dependent cell membrane repair augments antitumor immune response
Source: Nat Commun. 2022 Oct 24;13:6321. doi: 10.1038/s41467-022-34036-8 (PMC9592600; doi:10.1038/s41467-022-34036-8)
Supplement: Supplementary file 2 — Reporting Summary [file 41467_2022_34036_MOESM2_ESM.pdf]

nature portfolio

Corresponding author(s): Quanyin Hu

Last updated by author(s): Oct 5, 2022

Reporting Summary

Nature Portfolio wishes to improve the reproducibility of the work that we publish. This form provides structure for consistency and transparency in reporting. For further information on Nature Portfolio policies, see our Editorial Policies and the Editorial Policy Checklist.

Statistics

For all statistical analyses, confirm that the following items are present in the figure legend, table legend, main text, or Methods section.

n/a	Confirmed	
☐	☒	The exact sample size (n) for each experimental group/condition, given as a discrete number and unit of measurement
☐	☒	A statement on whether measurements were taken from distinct samples or whether the same sample was measured repeatedly
☐	☒	The statistical test(s) used AND whether they are one- or two-sided<br>*Only common tests should be described solely by name; describe more complex techniques in the Methods section.*
☐	☒	A description of all covariates tested
☐	☒	A description of any assumptions or corrections, such as tests of normality and adjustment for multiple comparisons
☐	☒	A full description of the statistical parameters including central tendency (e.g. means) or other basic estimates (e.g. regression coefficient) AND variation (e.g. standard deviation) or associated estimates of uncertainty (e.g. confidence intervals)
☐	☒	For null hypothesis testing, the test statistic (e.g. F, t, r) with confidence intervals, effect sizes, degrees of freedom and P value noted<br>*Give P values as exact values whenever suitable.*
☒	☐	For Bayesian analysis, information on the choice of priors and Markov chain Monte Carlo settings
☒	☐	For hierarchical and complex designs, identification of the appropriate level for tests and full reporting of outcomes
☒	☐	Estimates of effect sizes (e.g. Cohen's d, Pearson's r), indicating how they were calculated

Our web collection on statistics for biologists contains articles on many of the points above.

Software and code

Policy information about availability of computer code

Data collection	NIS-Elements (Version 4.60), Attune NxT Flow Cytometer System, In vivo imaging system, Agilent 1220 Infinity system, Malvern Zetasizer Software version 7.11, i-control 2.0 system, FEI Tecnai T-12 Cryo TEM system
Data analysis	All statistical analyses were performed on Graphpad Prism (version 8). All flow cytometry data were analyzed on FlowJo software package (Version 10.4). Living image software (Perkin Elmer, version 4.5) was used to analyse bioluminescent and fluorescent images.

For manuscripts utilizing custom algorithms or software that are central to the research but not yet described in published literature, software must be made available to editors and reviewers. We strongly encourage code deposition in a community repository (e.g. GitHub). See the Nature Portfolio guidelines for submitting code & software for further information.

Data

Policy information about availability of data

All manuscripts must include a data availability statement. This statement should provide the following information, where applicable:

- Accession codes, unique identifiers, or web links for publicly available datasets
- A description of any restrictions on data availability
- For clinical datasets or third party data, please ensure that the statement adheres to our policy

The authors declare that all the data supporting the findings of this study are available within the article, and the Supplementary Information and Source Data file.

Field-specific reporting

Please select the one below that is the best fit for your research. If you are not sure, read the appropriate sections before making your selection.

- [x] Life sciences
- [ ] Behavioural & social sciences
- [ ] Ecological, evolutionary & environmental sciences

For a reference copy of the document with all sections, see nature.com/documents/nr-reporting-summary-flat.pdf

Life sciences study design

All studies must disclose on these points even when the disclosure is negative.

Sample size	The sample size were determined by G-power analysis software correspondingly.
Data exclusions	No data were excluded.
Replication	Experiments were repeated at least three time unless otherwise stated in the respective figure legend. Experimental findings were reproducible.
Randomization	All samples and cells are randomly allocated to each group. Mice were randomly allocated to each treatment group.
Blinding	The investigator is not blinded for most of the in vitro experiments since the experimental design, execution, and data analysis are performed by the same person. For the in vivo experiments such as bioluminescence imaging, tumor size measurement and survival monitoring were conducted by independent operators, who were blinded to the group allocation during data collection and analysis.

Reporting for specific materials, systems and methods

We require information from authors about some types of materials, experimental systems and methods used in many studies. Here, indicate whether each material, system or method listed is relevant to your study. If you are not sure if a list item applies to your research, read the appropriate section before selecting a response.

Materials & experimental systems

n/a	Involved in the study	
[ ]	[x]	Antibodies
[ ]	[x]	Eukaryotic cell lines
[x]	[ ]	Palaeontology and archaeology
[ ]	[x]	Animals and other organisms
[x]	[ ]	Human research participants
[x]	[ ]	Clinical data
[x]	[ ]	Dual use research of concern

Methods

n/a	Involved in the study	
[x]	[ ]	ChIP-seq
[ ]	[x]	Flow cytometry
[x]	[ ]	MRI-based neuroimaging

Antibodies

Antibodies used

The following primary antibodies were used for flow cytometry. They are listed as antigen first, followed by supplier, catalog number and clone/lot number as applicable.
1) Anti-mouse CD45, Biolegend, cat. no. 103108, Clone: 30-F11;
2) Anti-mouse CD11b, Biolegend, cat. no. 101227, Clone: M1/70;
3) Anti-mouse IFNγ, Biolegend, cat. no. 505806, Clone: XMG1.2;
4) Anti-mouse F4/80, Biolegend, cat. no. 123116, Clone:BM8;
5) Anti-mouse Granzyme B, Biolegend, cat. no. 372212, Clone: QA16A02;
6) Anti-mouse CD3, Biolegend, cat. no. 100236, Clone: 17A2;
7) Anti-mouse CD4, Biolegend, cat. no. 100406, Clone: GK1.5;
8) PE Anti-mouse CD8a, Biolegend, cat. no. 100708, Clone: 53-6.7;
9) Anti-mouse CD11b, Biolegend, cat. no. 101206, Clone: M1/70;

The following primary antibodies were used for ELISA. They are listed as antigen first, followed by supplier, catalog number and clone/lot number as applicable.
1) Anti-rat IgG, eBioscience, cat. no. 88-50490-22;
2) Anti-mouse TNF-α, Biolegend, cat. no. 430904;
3) Anti-mouse IFN gamma, Biolegend, cat. no. 430804;

The following antibodies were used for western blot. They are listed as antigen first, followed by supplier, catalog number and clone/lot number as applicable.

1) HRP Anti-beta Actin antibody [AC-15], Abcam, cat. no. ab49900;
2) Gasdermin D (E9S1X) Rabbit mAb, Cell Signaling Technology, cat. no. 39754S;
3) Cleaved Gasdermin D (Asp276) (E3E3P) Rabbit mAb, Cell Signaling Technology, cat. no. 10137S
4) Cleaved Caspase-1 (Asp296) (E2G2I) Rabbit mAb, Cell Signaling Technology, cat. no. 89332S
5) HMGB1 (D3E5) Rabbit mAb, Cell Signaling Technology, cat. no. 6893S
6) Anti-rabbit IgG, HRP-linked Antibody, Cell Signaling Technology, cat. no. 7074P2

Validation: All antibodies are commercially available. Antibodies employed here in our manuscript were previously reported and routinely used for the application used. The validation and quality control are performed by the corresponding vendors.

Eukaryotic cell lines

Policy information about cell lines

Cell line source(s)	The murine melanoma cell line B16F10 and murine breast cancer cell line 4T1 are from ATCC. The murine B16F10-luc and 4T1-luc are purchased from Imanis Life Sciences. ID-8-Luc cells are provided by Dr. Paula Hammond's lab at MIT.
Authentication	The cell lines were morphologically confirmed according to the information provided by ATCC and Imanis Life Sciences.
Mycoplasma contamination	All cell lines are tested for mycoplasma contamination. No mycoplasma contamination is found.
Commonly misidentified lines (See ICLAC register)	No commonly misidentified cell lines were used.

Animals and other organisms

Policy information about studies involving animals; ARRIVE guidelines recommended for reporting animal research

Laboratory animals	Male and female C57BL/6 mice (6-8 weeks), and female Balb/c mice (6-8 weeks) were purchased from Jackson Lab. The animals were bred in the pathogen-free facility with a 12 h light/dark cycle at 20 ± 3 °C and humidity (40%-70%) and had ad libitum access to food and water.
Wild animals	The study did not involve wild animals.
Field-collected samples	The study did not involve samples collected from field.
Ethics oversight	The animal study protocol (M006373) was approved by the Institutional Animal Care and Use Committee at the University of Wisconsin-Madison.

Note that full information on the approval of the study protocol must also be provided in the manuscript.

Flow Cytometry

Plots

Confirm that:

- [x] The axis labels state the marker and fluorochrome used (e.g. CD4-FITC).
- [x] The axis scales are clearly visible. Include numbers along axes only for bottom left plot of group (a 'group' is an analysis of identical markers).
- [x] All plots are contour plots with outliers or pseudocolor plots.
- [x] A numerical value for number of cells or percentage (with statistics) is provided.

Methodology

Sample preparation	For tissue sample, the tissue was first mechanically disrupted from mice and divided into small pieces and homogenized in cold staining buffer to form single cell suspensions in the presence of digestive enzyme.
Instrument	ThermoFisher Attune Flow Cytometer
Software	FlowJo software package (version 10.4; TreeStar, USA, 2014)
Cell population abundance	No sorting was performed.
Gating strategy	The gating strategies are displayed in the supplementary information. Briefly, preliminary FSC/SSC gates were utilized for debris exclusion and locating the position of lymphocytes. Immune cells were gated by FITC/APC/SSA to specify dendritic cells or T cells. Activated dendritic cells were gated by CD80-PE/CD86-APC and activated T cells were gated by CD3-APC/CD8-PE.

- [x] Tick this box to confirm that a figure exemplifying the gating strategy is provided in the Supplementary Information.
